# The impact of interactive advertising on consumer engagement, recall, and understanding: A scoping systematic review for informing regulatory science

**DOI:** 10.1371/journal.pone.0263339

**Published:** 2022-02-03

**Authors:** Kristen Giombi, Catherine Viator, Juliana Hoover, Janice Tzeng, Helen W. Sullivan, Amie C. O’Donoghue, Brian G. Southwell, Leila C. Kahwati

**Affiliations:** 1 RTI International, Research Triangle Park, Durham, NC, United States of America; 2 Office of Prescription Drug Promotion, Center for Drug Evaluation and Research, US Food and Drug Administration, Silver Spring, Maryland, United States of America; University of Oklahama Norman Campus: The University of Oklahoma, UNITED STATES

## Abstract

We conducted a scoping systematic review with respect to how consumer engagement with interactive advertising is evaluated and if interactive features influence consumer recall, awareness, or comprehension of product claims and risk disclosures for informing regulatory science. MEDLINE, PsycINFO, Business Source Corporate, and SCOPUS were searched for original research published from 1997 through February 2021. Two reviewers independently screened titles/abstracts and full-text articles for inclusion. Outcomes were abstracted into a structured abstraction form. We included 32 studies overall. The types of interactive ads evaluated included website banner and pop up ads, search engine ads, interactive TV ads, advergames, product websites, digital magazine ads, and ads on social network sites. Twenty-three studies reported objective measures of engagement using observational analyses or laboratory-based experiments. In nine studies evaluating the association between different interactivity features and outcomes, the evidence was mixed on whether more interactivity improves or worsens recall and comprehension. Studies vary with respect to populations, designs, ads evaluated, and outcomes assessed.

## 1. Introduction

In 2020, it is estimated that nearly $356 billion was spent on digital advertising in the United States [1]. Much of this advertising consists of display ads, social media ads, search engine marketing, and email marketing often with interactive components to target the 85% of US adults who go online daily [2]. An interactive ad encourages consumers to interact with the ad (and thus the brand), rather than just passively view the ad. Although interactivity is often considered a vital element of successful online advertising [3, 4], its impact on consumer engagement and decision-making is not entirely clear.

The academic definition of interactive advertising has evolved and varied at least in part as possibilities for ad design and placement have shifted, meaning interactive advertising can be defined differently depending on the context. Experts have defined interactive ads in terms of processes, features, and/or user perceptions, and no consensus about the definition has been reached to date [5–14]. Conceptual frameworks considered by researchers in approaching interactive advertising have tended to include descriptions of how users behave in response to ads [13, 15–17]. Metrics employed by the advertising industry also have shifted over time. The operationalization of interactive advertising often has been determined by the conceptual framework used and the outcome of interest to the researcher.

With an increased presence of interactive advertising in digital and social media [18], it is critical to understand how consumers engage with these types of advertisements and whether interactive features influence consumer recall, awareness, or comprehension of product claims and risk disclosures. This is of particular importance for products or services for which advertising content is regulated, such as prescription drugs, alcohol, tobacco, and financial products or services, to ensure that such advertising does not introduce barriers or challenges to consumer understanding of risks associated with such products. Especially within the past decade, regulatory science researchers have embraced the tools of social science to assess consumer perceptions of risk as well as potential impediments to consumer understanding [19, 20]. Social science research can offer evidence of advertising effects on consumer perceptions, and agencies such as the U.S. Food and Drug Administration have used such approaches to assess consumer engagement with different types of advertisements, such as direct-to-consumer prescription drug television ads [21]. In order to assess whether interactive advertising poses new theoretical challenges or opportunities, we conducted a scoping systematic review to summarize the research related to consumer engagement with interactive advertisements and impact on recall and understanding of product claims and risk disclosures.

## 2. Methods

The protocol for this scoping review was registered at Open Science Framework on October 26, 2020 [22]. The goal of this scoping systematic review was to describe the extant literature on interactive advertising and consumer engagement, particularly as it concerned regulated product advertising and its influence on comprehension of product claims and risk disclosures. We designed the four research questions (RQs) that guided this scoping review to identify gaps in the evidence base and summarize important considerations needed to inform the design and conduct of future primary research studies in this area. The four RQs were:

RQ 1: What methods and measures are used to evaluate consumer engagement with interactive advertisements in empirical studies?
RQ 2: In empirical studies of interactive advertising in naturalistic or real-world contexts, to what extent do consumers engage with interactive advertisements?
RQ 3: What is the association between features of interactive advertisements for goods or services and consumer engagement, recall, awareness, or comprehension of product claims and risk disclosures?
RQ 4: How do interactive advertisements for goods and services compare to non-interactive advertisements (e.g., traditional print or broadcast advertisements) with respect to consumer engagement, recall, awareness, and comprehension of product claims and risk disclosures?

### 2.1 Search and data sources

We searched MEDLINE via PubMed, PsycINFO, Business Source Corporate, and SCOPUS for original research published in English from January 1, 1997, through February 17, 2021, using search terms related to advertising and marketing, internet, and the outcomes of interest (e.g., engagement, knowledge, click-through rate). Little research on digital advertising was conducted prior to the mid-1990s, and our preliminary evidence scan showed very few papers published prior to 1997. The detailed search strategy is in S1 Appendix. We also searched reference lists of systematic and narrative reviews and editorials where relevant.

### 2.2 Study selection

Two reviewers independently screened titles/abstracts and full-text articles for inclusion based on study selection criteria for each research question. Disagreements at the full-text review stage were resolved by a third reviewer. Detailed study selection criteria are described in S2 Appendix. In brief, we included all studies among persons of any age in the general public who were characterized as being a potential consumer target for interactive advertising. For all RQs, we included studies that examined exposure to interactive advertisements, which we defined as the promotion of a product, service, or idea using various features or tools that provide the opportunity for persons to interact directly with the ad and potentially influence/inform the remaining sequence, appearance, or content to be presented about the product, service, or idea. For RQ 2, we included only studies with exposure to interactive advertising in naturalistic or real-world contexts. For RQ 3, studies that compared alternative versions of advertisements with interactive elements that varied with respect to the type or level of interactivity were selected. For RQ 4, studies that compared interactive advertisements with traditional advertising (i.e., print ads, broadcast ads, or online/internet ads without interactive elements) were included.

Eligible outcomes varied by RQ. For RQ 1, we included studies with any measure of consumer engagement. For RQ 2, we required objective measures of engagement such as time spent viewing, content navigation, click-through rates, page views, shares, likes, or leaving comments. For RQs 3 and 4, we required studies to report outcomes including consumer recall, awareness, and comprehension of product claims, risk disclosures, or both. Lastly, we included only studies conducted in countries designated as *very highly developed* per the United Nations Human Development Index to maximize applicability to decision-makers in such settings [23].

### 2.3 Data abstraction and synthesis

For each article included, one reviewer abstracted relevant study characteristics and outcomes into a structured abstraction form, and a second senior reviewer checked the form for completeness and accuracy. We narratively synthesized findings for each RQ by summarizing the characteristics and results of the included studies in narrative and tabular formats. Because this was designed as a scoping review, we did not conduct risk of bias assessments on included studies, quantitatively synthesize findings, or conduct strength of evidence assessments.

## 3. Results

**Fig 1:**
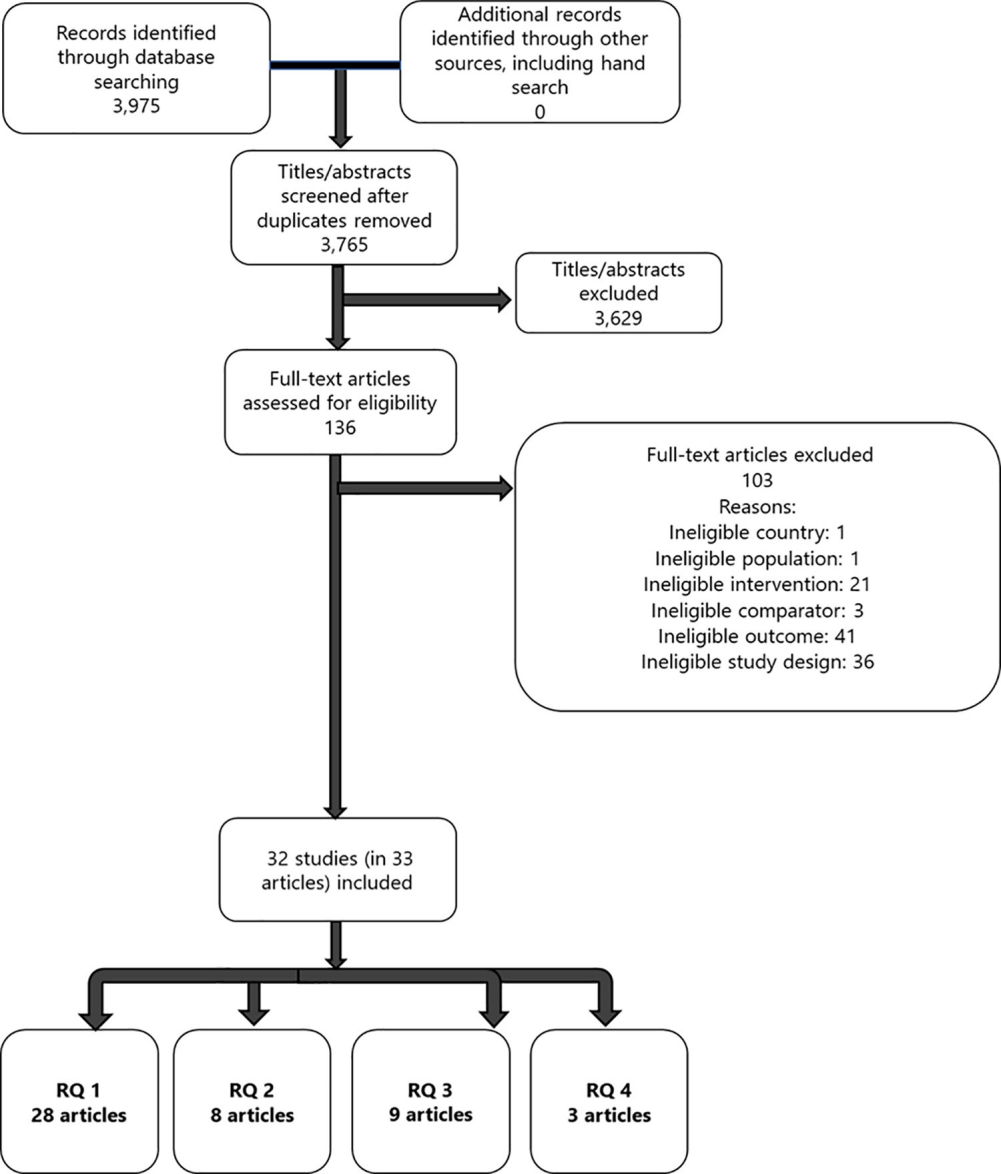
Preferred reporting items for systematic reviews and meta-analyses literature flow diagram.

We screened 3,765 titles and abstracts and 136 full-text articles. We included 32 studies published in 33 articles (Fig 1) [7, 24–55]. Twenty-three studies addressed RQ 1, eight studies addressed RQ 2, nine studies addressed RQ 3, and four studies addressed RQ 4. An overview of included studies is provided in Table S4-1 in S4 Appendix. A list of full-text studies that we reviewed and excluded is provided in the S3 Appendix.

### 3.1 Research question 1: What methods and measures are used to evaluate consumer engagement with interactive advertisements in empirical studies?

#### 3.1.1 Study characteristics

We identified 23 studies eligible for RQ 1 that were published between the years 1997 and 2019 and conducted across multiple countries [24–30, 33, 35–42, 46, 47, 50–53]. An overview of the studies is in Table S4-1 and S4-2 in S4 Appendix. Six were observational studies evaluating consumer response to real-world advertisements or campaigns [24, 25, 28–30, 37]. The rest of the studies were experiments conducted in laboratory or controlled environments. The sample sizes across the included studies ranged from 20 to 116,168 participants; however, two studies [29, 30] did not report the number of persons participating in the study.

The types of interactive advertisements evaluated varied across the included studies. Six studies [26, 33, 35, 40, 47, 50] evaluated banner ads, three studies [7, 36, 46] evaluated product websites, three studies [29, 30, 41] evaluated paid search engine ads, three studies [38, 51, 52] evaluated interactive television ads, two studies [24, 27] evaluated social network site ads, one study [39] evaluated a pop-up ad and the rest of the studies evaluated other types of digital ads. This included short-message-service TV marketing [37], an ad with a video clip embedded in a digital magazine [42], ads within a simulated online store [53], and combinations of different types of digital and online ads [25, 30]. The type of products advertised across the included studies included unregulated consumer products (e.g., digital cameras) and services (e.g., travel planning); regulated products and services (car insurance, financial); and health/health behavior campaigns.

#### 3.1.2 Findings

**Fig 2:**
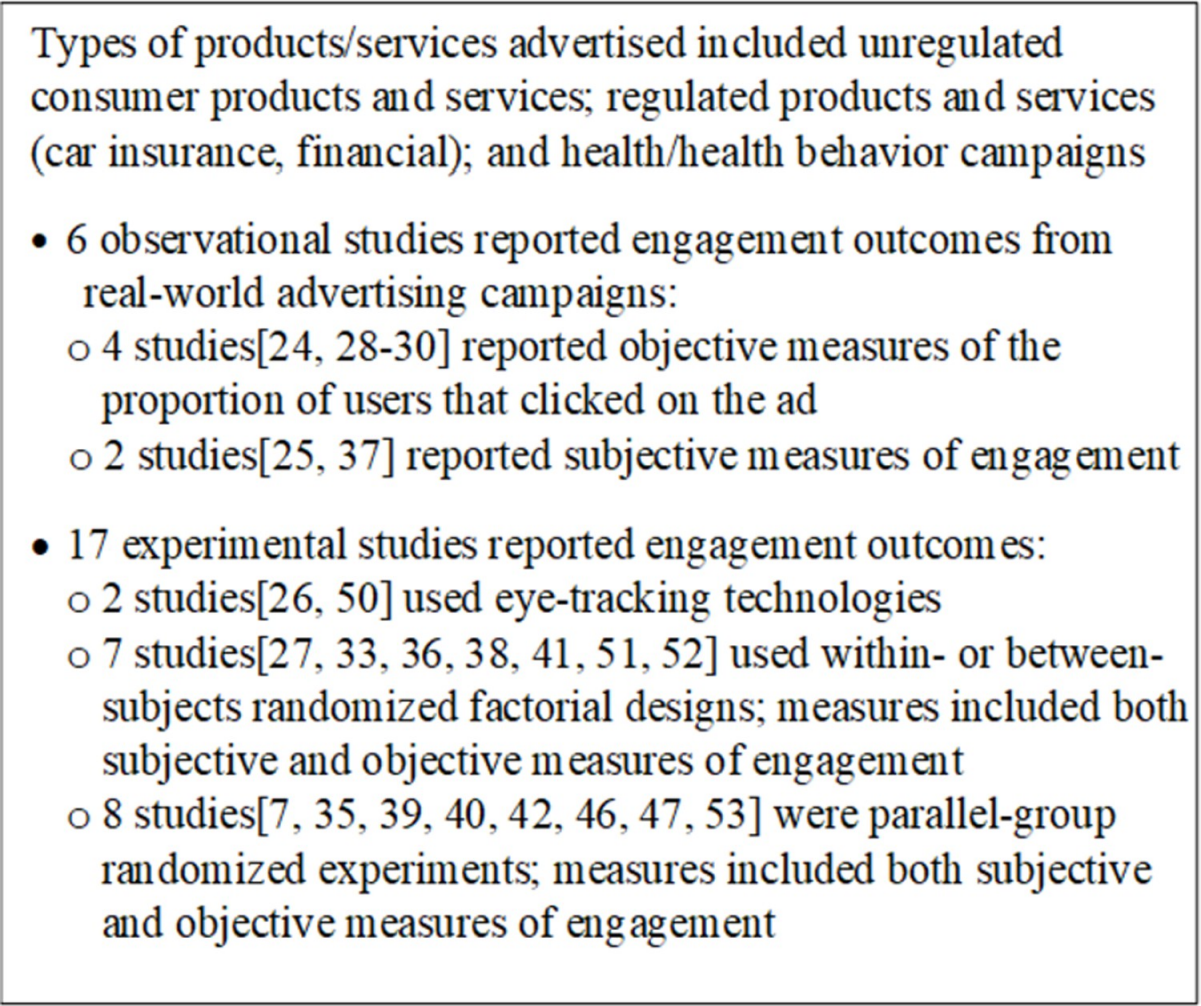
Summary of findings from 23 studies addressing research question 1.

An overview of findings is in Fig 2. Authors of the six observational studies reported engagement outcomes associated with real-world advertising or marketing campaigns [24, 25, 28–30, 37]. Authors of four studies reported objective measures of the proportion of users exposed to an ad that clicked on the ad (i.e., “click-through rates”) by using platform-specific (e.g., Facebook, Google AdWords) analytic tools for advertisers [24, 29], specialized web tracking software that members of a market research panel consented to have installed on their computers to monitor web behavior [28], or a unique event identifier created on the advertiser’s server whenever an online ad was clicked [30]. Authors of the other two observational studies reported subjective measures of engagement. In one study, authors used audio, computer-assisted self-interviews that asked respondents about their engagement with online marketing of a specific class of product [25]. In the other study, authors used post-campaign surveys (mode not specified) to evaluate engagement outcomes [37].

Authors of the 17 experimental studies reported engagement outcomes from experiments using actual real-world ads or from experiments using fictitious ads designed specifically for the experiment. Authors of the experimental studies controlled participant exposure to the ads, and depending on the measure, outcome measurement occurred either concurrently with the ad exposure or through completion of post-exposure surveys or interviews.

Two of the experimental studies used objective measures of ad engagement employing eye-tracking technologies during exposure to evaluate user engagement with digital ads placed on online platforms (Facebook page, blog, and industry-specific search engine) [26, 50]. In Muñoz-Leiva, Hernández-Méndez, and Gómez-Carmona [26] the ads used were fictitious, and the sites they were placed on were mocked up to resemble existing platforms (e.g., Facebook). In Barreto [50], each participant’s own Facebook page and the authentic Facebook page for a specific brand of athletic shoe was used. In both studies, authors first calibrated the eye-tracking equipment for each participant, then assigned one or more tasks for the participants to complete (e.g., navigate to find a specific item). The eye-tracking technology measured fixation counts and duration of fixation on the ad portion of the screens as participants navigated through the task.

Seven of the experimental studies were designed using a within- or between-subjects randomized factorial design or both [27, 33, 36, 38, 41, 51, 52]. In these studies, authors manipulated two or more ad features, including message/information content, tone, amount, or presentation order; images; screen placement; and level of interactivity. Eight of the experimental studies were parallel-group randomized experiments with one group assigned to a manipulated ad exposure in one or more ways and the other group assigned to a control ad exposure [7, 35, 39, 40, 42, 46, 47, 53]. In both types of experimental studies, measures of ad engagement varied and included both subjective (e.g., user intentions as to whether they would click the ad or like or share the ad post) and objective measures (e.g., actual click-through rates on ads encountered, view duration tracked by computer). Nearly all studies also measured additional outcomes such as attitudes toward ads, ad or brand recall, or purchase intentions through post-exposure surveys.

### 3.2 Research question 2: In empirical studies of interactive advertising in naturalistic or real-world contexts, to what extent do consumers engage with interactive advertisements?

#### 3.2.1 Study characteristics

Eight studies addressed RQ 2; these were published between 2006 and 2019 (Table S4-3 in S4 Appendix) [24, 28–31, 39, 47, 54]. Six were observational studies [24, 28–31, 54], and two studies were experimental but conducted in real-world (i.e., not laboratory) settings [39, 47]. The sample sizes across the included studies ranged from 30,638 to 2,000,000 participants. The types of interactive advertisements evaluated varied and could include more than one type of ad. Three studies evaluated banner ads [28, 30, 47], two studies evaluated social network site ads [24, 31], and one study evaluated a pop-up ad [39]. Three studies evaluated other types of digital ads including paid search engine ads and video ads [28, 29, 54]. The type of products advertised across the included studies included unregulated consumer products and services and health/health behavior campaigns.

Authors measured consumer engagement with click-through rates; page views; and/or number of “likes,” comments, or shares on social media. The two experimental studies analyzed click-through rates for banner and pop-up ads [39, 47], while the six observational studies analyzed click-through rates for banner ads [28, 30], search ads [28–30, 54], and social media interaction [24, 31].

#### 3.2.2 Findings

**Fig 3:**
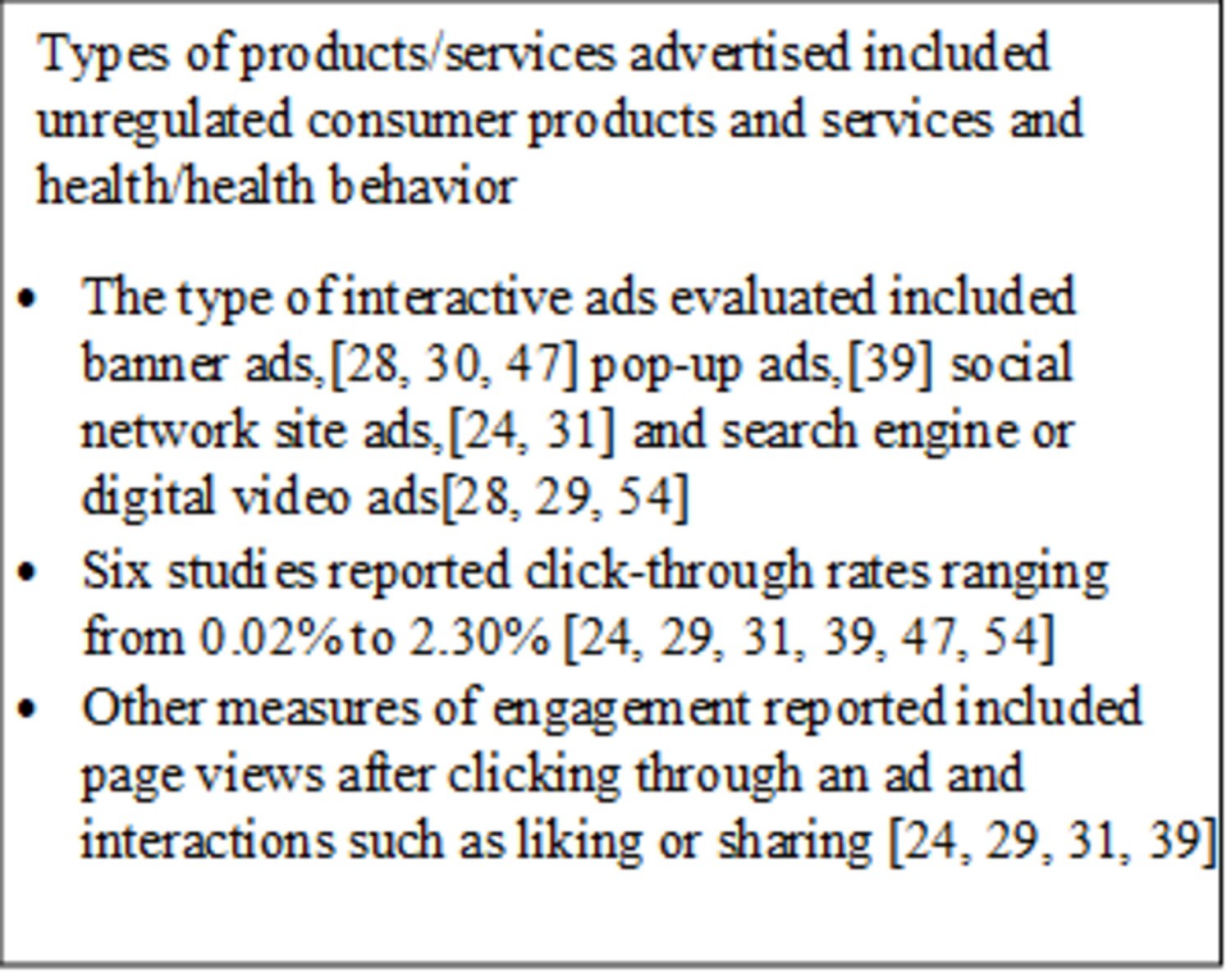
Summary of findings from 8 studies addressing research question 2.

An overview of findings is in Fig 3. The level of engagement by consumers varied across studies. Six studies reported click-through rates ranging from 0.02% to 2.30% [24, 29, 31, 39, 47, 54]. Two of these studies also reported differences in click-through rates when selected characteristics of the ad were varied, such as differences on which page the ad was placed, a variable delay before the ad was displayed [39], or whether the ads were static or morphing and whether they were context matched to the website on which they were placed [47]. In contrast to other studies reporting click-through rates, Graham et al. [30] reported a much higher click-through rate (81.6%); this study used ads to recruit individuals to a website to register for smoking cessation treatment.

Other measures of consumer engagement beyond click-through rates included number of page views (after clicking through an ad) and interactions such as liking, sharing, or posting comments to ads on social networking platforms. Two studies measured page views, which is the number of pages the viewer visited after going to the landing site [29, 39]. In Birnbaum et al. [29] the median number of pages visited on the website (not including other relevant websites that were linked on the study site) was 1.29. Moe [39] measured the difference in number of page views when users were exposed to the ad on a gateway page of an informational website compared with exposure to the ad from a content page of the website. The mean number of page views after an ad on a content page (6.31) was higher than page views after an ad on a gateway page (4.86, *P* < .001), suggesting greater engagement from consumers when involved in the content.

Two studies measured interactive engagement with social media ads through “likes” and shares [24, 31]. Horrell et al. [24] defined levels of consumer engagement as “low” if a consumer liked a page or reacted to a post and “medium” if a consumer shared or commented on a post. Over a 5-week advertising campaign targeted to 91,385 users of a specific Facebook page site targeting lung cancer awareness, the page had 2,602 reactions to posts, 149 page likes, 452 shares, and 157 comments [24]. Similarly, Platt et al. [31] reported findings from a 1-month time period in which a Michigan biobank advertising campaign was targeted to an estimated 2 million state residents aged 18 to 28. The campaign’s social media presence garnered 516 page likes, 477 ad likes, 25 page post shares, and 30 entries into an advertised photo contest. This study also reported that a greater percentage of viewers clicked an ad or post they saw when it was associated with the name of a friend who had already liked the Facebook page [31].

### 3.3 Research question 3: What is the association between features of interactive advertisements for goods or services and consumer engagement, recall, awareness, or comprehension of product claims and risk disclosures?

#### 3.3.1 Study characteristics

We identified nine studies eligible for RQ 3 that were published between the years 1997 and 2019 (Tables S4-4 and S4-5 in S4 Appendix) [26, 32, 34, 36, 43–45, 51, 53]. Eight studies were conducted as experiments [26, 32, 34, 36, 43, 44, 51, 53], and the remaining study was a meta-analysis [45]. The sample sizes across the included primary research studies ranged from 60 to 1,600 participants. The type of advertisements evaluated varied. Four studies [32, 34, 36, 44] evaluated product websites, one study [26] evaluated banner ads, one study [43] evaluated both banner ads and advergames, and two studies [51, 53] evaluated other types of digital ads (e.g., interactive TV ads and interactive ads in a simulated online store). The included studies manipulated the ad stimuli to vary the level of interactivity or the type of interactive features included in the ad. Interactive features used in these studies included clickable hyperlinks, navigation bars, navigation buttons, rollover and clickable animation, responsive chat features, comment forms, and interactive game elements. The type of products advertised across the included studies included unregulated consumer products and services as well as regulated products or services.

The meta-analysis reported on 63 experimental studies (total N = 13,484) that evaluated how web interactivity affects various psychological outcomes and how those effects are moderated [45]. Of the included studies, half focused on interactivity within an advertising context, and 25% reported cognition outcomes, the only outcomes of relevance to this review.

#### 3.3.2 Findings

**Fig 4:**
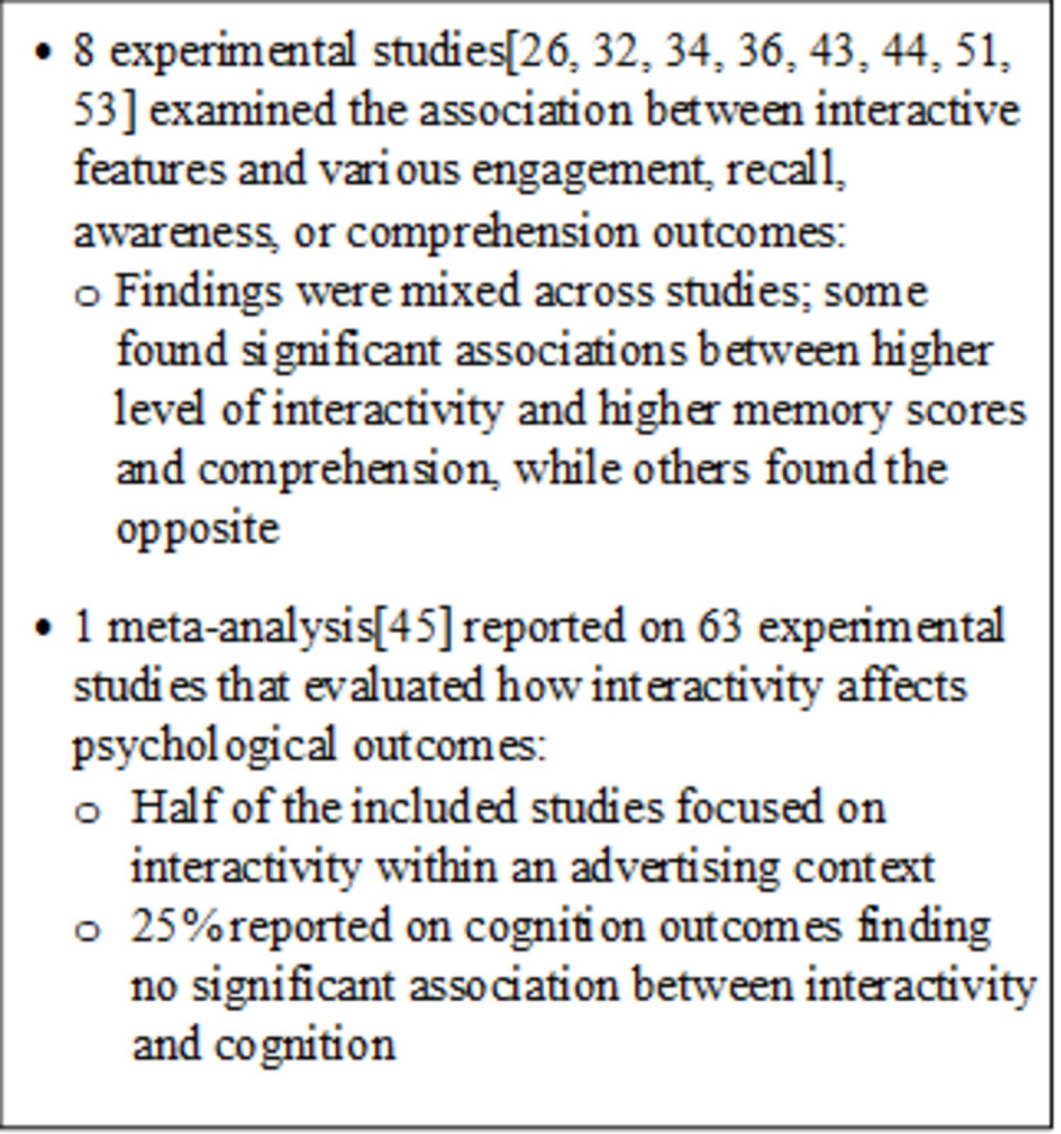
Summary of findings from 9 studies addressing research question 3.

An overview of findings is in Fig 4. In the meta-analysis, Yang and Shen [45] defined cognition measures such as comprehension, elaboration, knowledge acquisition, and recall. The authors reported no significant association between interactivity and cognition (correlation coefficient 0.05, *P* = .25). Across the eight primary research studies for this RQ, outcomes varied widely by level or type of interactivity. Five of the studies measured consumer recall of the brand, product, or service advertised [32, 34, 36, 43, 44]. Four of these involved websites or web pages with varying levels of interactivity [32, 34, 36, 44].

In Chung and Zhao [36], undergraduate university students viewed websites advertising cameras, which were classified as either low, medium, or high interactivity based on the number of hyperlinks included. They found a significant association between a higher number of clicks available and higher memory scores [36].

In Chung and Ahn [32], authors asked participants to view either a website with a linear structure (scroll to bottom of page and click link to move to next page), an interactive structure (multiple links available on the page), or a mixed linear and interactive structure and asked them to write down all the product information they could recall after exposure [32]. The authors found that participants who viewed the linear web page exhibited the highest memory score [32].

In Macias [44], participants viewed either a low or high interactivity website that advertised one of two consumer products. The high interactivity websites included rollover animation, hyperlinks, comment forms, and chat features. The authors found that participants who viewed the high interactivity website exhibited greater comprehension [44].

Polster et al. [34] reported the results of a study comparing interactive and noninteractive versions of a website with important safety information (ISI) about a fictitious medication viewed either on a desktop computer or smartphone. Authors found that a higher percentage of participants allocated to noninteractive websites saw any ISI as measured through objective clicking and scrolling behavior compared with participants who were allocated to the interactive websites (*P* < .001). Further, a higher proportion of desktop-using participants allocated to noninteractive websites recalled at least one relevant side effect compared with participants allocated to the interactive websites (*P* < .001) [34]. A higher proportion of participants using a smartphone allocated to noninteractive websites also had higher recall of at least one relevant side effect compared with participants who were allocated to interactive sites, but this finding was only statistically significant for one of the two noninteractive layouts [34]. Authors also reported the mean percentage correct recognition of medication side effects and conducted additional analyses of recognition limited to those participants who saw any ISI (Table S4-4 in S4 Appendix).

Finally, in Daems et al. [43], Belgian secondary students viewed ads for a fictitious smartphone that were either interactive advergames, static in-game ads, interactive banner ads, or noninteractive banner ads. Authors found that interactive banner ads led to the highest percentage of participants exhibiting brand recognition (60.4%), followed by static in-game ads (22.4%), noninteractive banner ads (21.3%), and finally advergames (14.3%) [43]. They also found that interactive banner ads led to the highest memory of product characteristics (8.22 out of a 12-point scale), while noninteractive banner ads led to the lowest memory (3.87) [43].

Three studies measured time spent viewing ads and results were mixed [26, 51, 53]. In Cauberghe and De Pelsmacker [51], participants from a Belgian market research firm watched a Dutch travel agency interactive TV ad with low, medium, or high interactivity. The interactivity level varied based on the presence of clickable links, navigation bars, and two-way communication. The authors reported significantly more time spent viewing the high interactivity ad (6.1 minutes) than the low interactivity ad (4.4 minutes) [51]. In Yang [53], each participant viewed one interactive ad and one noninteractive ad for one of two consumer products. The high interactivity ads offered more user control over order of information, duration of each page, and ability to skip information. Authors found that interactive ads were viewed for less time than noninteractive ads (*P* < .01) [53]. In Muñoz-Leiva et al. [26], the authors compared “Travel 2.0 websites” with embedded vertical banner ads on 3 different platforms: a Facebook page, a blog, and a Tripadvisor page that varied by level of interactivity [26]. While the banner ads on all three platforms included a call to action and a clickable link to an airline website, the Facebook ad was the most interactive with the ability to like, comment, and share the ad followed by the blog with the ability to comment on the blog post and finally the Tripadvisor page. The authors used eye-tracking technology to measure the number of visual fixations on the ad, number of seconds until the first fixation on the ad, and total duration of fixations on the ad. They found a significant difference in the number of ad fixations (Facebook, 19.1; blog, 11.7; Tripadvisor (6.1), *P* < .001). Significant differences were also observed across platforms for other measures (Table S4-5 in S4 Appendix) [26].

### 3.4 Research question 4: How do interactive advertisements for goods and services compare with noninteractive advertisements (e.g., traditional print or broadcast advertisements) with respect to consumer engagement, recall, awareness, and comprehension of product claims and risk disclosures?

#### 3.4.1 Study characteristics

We identified three studies eligible for RQ 4 that were published between the years 2008 and 2018 (Table S4-6 in S4 Appendix) [30, 48, 49]. One was an observational study [30], and the other two studies were conducted as experiments. The sample sizes for the two experiments were 233 [49] and 9,902 [48] participants; the observational study [30] did not report the number of persons evaluated. The types of interactive advertisements varied. The observational study [30] compared banner ads and paid search ads (interactive advertising) with billboards, TV ads, radio ads, outdoor signage, direct mail, and physician detailing (noninteractive advertising). One experimental study [48] had print flyer, online flyer, and no flyer groups, while the other experimental study [49] compared a standard TV commercial, a PC advergame, and an interactive TV commercial offering an advergame.

Eligible outcomes for this review reported across the three included studies also varied. The observational study [30] evaluated outcomes associated with real-world advertising including the number of log-ins and pages viewed, session length, and long-term cookies. Authors of the two experimental studies [48, 49] randomized participants to different ad types and evaluated recall in addition to other outcomes such as attitudes, which were not within the scope of this review.

#### 3.4.2 Findings

**Fig 5:**
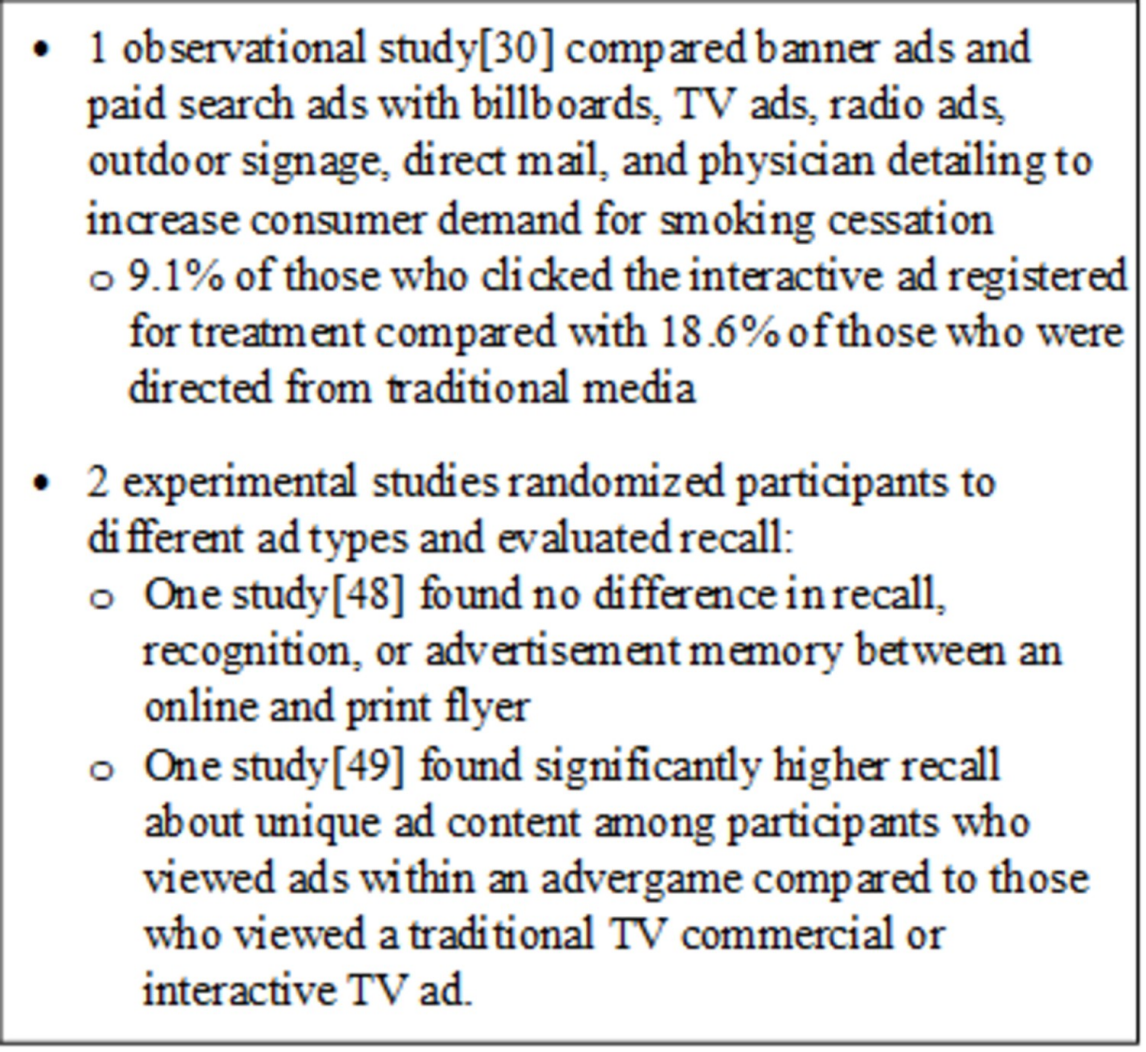
Summary of findings from 3 studies addressing research question 4.

An overview of findings is in Fig 5. Across the three included studies, outcomes varied widely. Graham et al. [30] examined how online advertising increases consumer demand for smoking cessation treatments in Minnesota and New Jersey (N = NR) by comparing the impact of interactive advertisements (banner ads, paid search ads) versus traditional advertisements (billboards, TV ads, radio ads, outdoor signage, direct mail, physician detailing). Outcomes related to engagement are reported in the RQ 2 section of this review. Ultimately, 9.1% of those who clicked the interactive ad registered for treatment compared with 18.6% of those who were directed to the website from traditional media [30]. The authors found that compared with traditional ads, online ads engaged a higher percentage of males, young adults, racial/ethnic minorities, individuals with a high school education or less, and dependent smokers. While the authors found significant differences in website engagement metrics (e.g., average session length, pages viewed, percentage posting in public forums) between online and traditional ad responders, they noted that the differences in utilization are too small in magnitude to be meaningful.

Ieva et al. [48] estimated the effect of an online versus print promotional advertising flyer on customer response with an experimental design recruiting from a random sample of customers from a supermarket chain (N = 9,902; however, only the 303 that reported viewing the flyer were included in the analysis). The online flyer was a replication of the print flyer with no banners, videos, or embedded links; however, users could click to zoom or move to another page. The authors found no statistically significant differences in recall, recognition, or advertisement memory measures between the online and print flyers.

Bellman et al. [49] compared the effectiveness of PC advergames, TV commercials, and interactive commercials enhanced with advergames on recall for four test brands of food or personal hygiene products in an experimental study. Members of an Australian audience panel (N = 233) were randomized to one of three ad types. The authors reported significantly higher unaided recall of at least three unique points about the ad content for participants who viewed the PC advergame compared with those who viewed the traditional TV commercial and as compared with the interactive TV ad. Authors observed no significant difference between participants who viewed the interactive TV commercial and the traditional TV commercial.

## 4. Discussion

### 4.1 Summary of evidence

Study design and outcomes varied widely within the evidence base for each RQ. That variation itself is noteworthy, as it affects comparability of results and suggests strengths and weaknesses of different approaches for future research in this arena. Through this review, we also can see ways in which existing literature may not yet be optimal for answering questions about consumer risk perception and decision making in response to interactive advertising; much available evidence focuses on indicators of short-term consumer attention in engaging with advertising more than on consumer information processing beyond eyeball movement or click behavior.

Within the 23 studies eligible for RQ 1 (which summarized methods and measures used to evaluate consumer engagement), six were observational studies and 17 were experimental studies. In the experimental studies, methods included within- and between-subjects randomized factorial design and parallel-group randomized experiments. In both types of studies, objective (e.g., click-through rates, eye-tracking metrics) and subjective (e.g., post-campaign surveys, interviews) measures were used to report engagement outcomes. This variability in methods is understandable. Some measures of engagement are most optimally assessed with experimental designs that allow control over content and resource-intensive measurement of respondents (e.g., eye-tracking metrics). Observational studies nonetheless also can offer objective measures of engagement on a larger scale and without the generalizability concerns stemming from volunteer bias inherent to small sample-sized experimental designs. We also did find examples of large-scale experiments [39, 42] involving manipulation of advertising stimuli conducted with various kinds of media (digital magazine, websites publishing reviews, news, or information).

Based on this review, consumer engagement is an umbrella concept covering a range of operationalization efforts. The ways in which studies measured engagement reflect 1) varying levels of technologic sophistication of the advertising platform or ad itself, 2) the salience of click-through rates as a metric in commercial advertising (regardless of the theoretical value of that metric to understanding consumer decision making), and 3) varying levels of integration into a broader social media campaign. We did not identify any differences in the way engagement was measured for regulated versus non-regulated products in this scoping review, per se, but the number of studies focused on regulated products or services also was quite limited. Future research on consumer engagement with interactive ads for regulated products should be able to use both observational or experimental designs, depending on the specific outcomes in question.

For RQ 2, eight identified studies reported on the extent to which consumers engage with interactive advertisements in naturalistic or real-world contexts. Consumer engagement was measured with click-through rates; page views; and/or number of “likes,” comments, or shares on social media. Click-through rate was the most common engagement measure used for this RQ; however, the way in which click-through-rates were calculated varied, limiting direct head-to-head comparisons across studies. A click-through rate may offer a conceptually simple way of measuring consumer engagement because it is closely aligned to the evaluation of cost-per-thousand advertisement impressions (i.e., cost-per-mille) and cost-per-click advertising campaigns. In practice, however, variability in click-through rate calculation limits the ability of current literature to offer definitive conclusions related to the concept. Moreover, in the context of evaluating regulated advertising, crude click-through-rates of a single hyperlink in a digital ad may not be enough to provide a nuanced understanding of whether users engage with specific parts of an ad, specifically, claims of benefits, risk disclosures, or both.

For RQ 3, we identified nine studies, eight of which were experiments, that focused on the association between features of interactive ads and consumer engagement, recall, awareness, or comprehension of product claims and risk disclosures. The studies varied the type or level of interactivity in the ad. Some studies found significant associations between higher levels of interactivity and higher memory scores, comprehension, and brand recognition. Other studies found the opposite: better recall and higher memory scores with fewer interactive features. Studies that measured time spent viewing the ads also had mixed results: one study found higher levels of interactivity led to more time spent viewing the ad, whereas one study found the opposite. Further, a meta-analysis reported no correlation between interactivity and measures of cognition.

The evidence for clear relationships between interactive features and outcomes of interest for this scoping review was mixed, precluding any definitive conclusions. Further, some studies addressing this RQ were published during an early era of online advertising that has faded in relevance to present circumstances. Importantly, we also found instances of confounding. In addition to manipulating interactivity, advertisers often manipulated other aspects of the ad not related to interactivity (e.g., tone, text or graphic content). Previous studies have demonstrated that for regulated products, such as prescription drugs, these features moderate consumer understanding of product claims and risk disclosures [21, 56]. Thus, future studies evaluating variations in interactive ads of regulated products and services should ensure that study designs and ad manipulations are robust for evaluating independent effects and potential interactions.

For RQ 4, three identified studies compared interactive with noninteractive advertisements with respect to consumer engagement, recall, awareness, and comprehension of product claims and risk disclosures. One observational study found that compared with traditional ads, online ads engaged certain segments of the population better. The two experimental studies found no significant differences between the interactive and traditional ads, but one study found significantly higher unaided recall for participants who viewed a PC advergame compared with those who viewed the traditional or the interactive ads. With the mixed results from this limited number of heterogeneous studies, there is no conclusive evidence on how interactive advertisements compare with noninteractive advertisements with respect to consumer engagement, recall, awareness, and comprehension of product claims and risk disclosures. The limited number of studies may reflect the challenge in conducting direct comparisons of traditional and interactive advertising in the same study. Digital and online advertising offer new and, in some cases, more objective ways of measuring advertising effectiveness that have no counterpart in the evaluation of traditional advertising. Given shifts away from traditional advertising to digital and online marketing because of better returns on investment and ability to target audiences, comparing traditional to interactive ads may not be a relevant comparison for future studies.

### 4.2 Limitations of evidence

Studies were quite heterogenous with respect to study design, populations evaluated, types of ads used, and measures reported; this limited our ability to conduct a robust synthesis of outcomes. Many studies were conducted among university students; whether findings from such studies would generalize to broader populations is not known. The measures used by some studies to evaluate product or service information recall or knowledge did not appear to be validated. The era over which studies were conducted was broad; some of the interactivity features or platforms used in included studies are likely obsolete or have been replaced by more sophisticated approaches to interactive advertising.

### 4.3 Limitations of this review

We limited this scoping review to studies published in English from very highly developed countries to increase applicability of findings to policy makers concerned with regulation of interactive advertising in such countries. Study indexing in bibliographic databases was variable and inconsistent; thus, it is possible we missed some relevant studies. Our RQs were focused on outcomes related to consumer engagement with interactive ads, and information recall and comprehension, as it related to product information or risk disclosures. We did not consider consumer attitudes or purchase behavior. We limited measures of engagement to studies conducted in naturalistic or real-world contexts because experimental studies typically manipulated ad exposure or instructed participants what to view and may have put limits on duration of exposure that would not reflect engagement outside of a controlled environment. We did not assess the risk of bias of included studies consistent with a scoping review approach.

### 4.4 Research gaps

Although the research on interactive advertising is extensive in terms of the volume of available publications, as judged by the size of our initial search yield, the amount of research specifically focused on the influence of interactive advertising on product information recall and specifically risk perception is sparse. Several studies that we screened but excluded as not eligible for this scoping review focused on evaluating tone, content, graphics, placement, or variable deployment of an interactive ad and impact on consumer attitudes about the product or brand or subsequent purchase intention or behavior (see S3 Appendix for a list of excluded studies). Whether such outcomes correlate to an accurate understanding of product features or services and risk disclosures is not known but could be relevant when considering interactive advertising for regulated products, such as prescription drugs, alcohol, tobacco, and financial products or services. Regardless, it is clear that available research on interactive advertising does not provide much of the evidence most useful to regulatory science focused on whether regulated advertising encourages informed decision making about products.

We need rigorously designed studies of consumer experiences with interactive advertising that use objective and validated measures to assess recall and understanding of product or service information and risk disclosures. We note a disjuncture between our selected studies and recent work on social media activities. A type of study we commonly encountered during screening but excluded as not eligible were studies evaluating the impact of influencer marketing through social media. Though not a focus of this scoping review, we noted many of these studies in the latter part of the time period we searched, suggesting an increasing use of this type of digital, interactive advertising for the future and a possible area for future inquiry.

## 5. Conclusion

This scoping systematic review summarized the research related to consumer engagement with interactive advertisements and impact on recall and understanding of product claims. The evidence shows that consumers do engage with interactive advertisements, but the evidence is mixed as to whether features of interactive advertising increase consumer engagement, recall, awareness, or comprehension of product claims and risk disclosures. Only a few studies compared traditional advertisements with interactive advertisements on these outcomes and these results also were mixed. Some of the limitations of existing interactive advertising literature as a source for informing regulatory science appears to reflect inconsistent labeling of concepts as well as adherence to industry metrics rather than regulatory science needs.

## Supporting information

S1 Checklist PRISMA checklist. (DOCX)

S1 Appendix Detailed search strategy. (DOCX)

S2 Appendix Study selection criteria. (DOCX)

S3 Appendix Excluded studies. (DOCX)

S4 Appendix Results tables. (DOCX)
