# Supplementary material for: The impact of interactive advertising on consumer engagement, recall, and understanding: A scoping systematic review for informing regulatory science
Source: PLoS One. 2022 Feb 3;17(2):e0263339. doi: 10.1371/journal.pone.0263339 (PMC8812936; doi:10.1371/journal.pone.0263339)
Supplement: S1 Appendix — (DOCX) [file pone.0263339.s002.docx]

## S1 Appendix. Detailed search strategy

**PubMed**

**January 1, 1997 through February 17, 2021**

**Advertising Terms**

#1 (“Advertising”[Mesh] OR “Direct-to-Consumer Advertising”[Mesh] OR “Direct-To-Consumer Screening and Testing”[Mesh] OR “Marketing”[Mesh] OR “Marketing of Health Services”[Mesh] OR “Public Service Announcements as Topic”[Mesh] OR “direct-to-consumer advertising”[Other Term] OR “direct-to-consumer promotion”[Other Term]) AND (“1997/01/01”[Date - Publication] : “3000”[Date - Publication]) Filters: English

**Internet Terms**

#2 (“Internet”[Mesh] OR “Social Media”[Mesh] OR “Smartphone”[Mesh] OR “Cell Phone”[Mesh] OR “Online Systems”[Mesh] OR “Internet”[Other Term] OR digital advertis*[Title/Abstract] OR online advertis*[Title/Abstract] OR digital market*[Title/Abstract] OR online market*[Title/Abstract] OR interactive advertis*[Title/Abstract] OR interactive market*[Title/Abstract] OR banner advertis*[Other Term] OR online advertis*[Title/Abstract] OR “Consumer Health Informatics”[Mesh] OR “Mobile Applications”[Mesh]) AND (“1997/01/01”[Date - Publication] : “3000”[Date - Publication]) Filters: English

**Measures and Outcomes Terms**

#3 (“Deception”[Mesh] OR “Disclosure”[Mesh] OR “Health Knowledge, Attitudes, Practice”[Mesh] OR “Health Behavior”[Mesh] OR “Consumer Behavior”[Mesh] OR “Safety”[Mesh] OR “Risk Assessment”[Mesh] OR “Information Seeking Behavior”[Mesh] OR “engagement”[Title/Abstract] OR “recall”[Title/Abstract] OR “knowledge”[Title/Abstract] OR attitude*[Title/Abstract] OR click-through-rate*[Other Term] OR “risk information”[Title/Abstract] OR “benefit”[Title/Abstract] OR risk*[Title/Abstract] OR success rate*[Other Term] OR “web behavior”[Title/Abstract]) AND (“1997/01/01”[Date - Publication] : “3000”[Date - Publication]) Filters: English

**Total**

#4 (#1 AND #2 AND #3) AND (“1997/01/01”[Date - Publication] : “3000”[Date - Publication]) Filters: English

**PsycINFO**

**January 1, 1997-February 17, 2021**

**Limit to peer reviewed journals**

**Advertising Terms:**

S1 DE “Advertising” OR DE “Digital Marketing” OR DE “Mass Media” OR DE “Marketing” OR TI “digital advertis*” OR AB “digital advertis*” OR TI “digital market*” OR AB “digital market*” OR TI “interactive advertis*” OR AB “interactive advertis*” OR TI “banner advertis*” OR AB “banner advertis*” OR TI “interactive market*” OR AB “interactive market*” OR KW “consumer information source” OR KW “Direct-to-Consumer Advertis*” Limiters - Publication Year: 1997-2020; Publication Type: Peer Reviewed Journal; English

**Internet Terms:**

S2 DE “Internet” OR DE “Smartphones” OR DE “Social Media” OR DE “Human Computer Interaction” OR DE “Digital Information” OR TI “digital platform*” OR AB “digital platform*” OR KW interactiv* W1 features OR TI interactiv* OR AB interactiv* OR KW website* OR KW Internet OR KW Computer* Limiters - Publication Year: 1997-2020; Publication Type: Peer Reviewed Journal; English

**Outcome and Measure Terms**

S3 DE “Consumer Behavior” OR DE “Consumer Attitudes” OR DE “Cognitive Processes” OR DE “Human Computer Interaction Measures” OR DE “Consumer Research” OR DE “Consumer Psychology” OR DE “Attitudes” OR SU “Recall (Learning)” OR DE “Retention” OR DE “Sentiment Analysis” OR DE “Decision Making” OR KW “Consumer Choices” OR KW “consumer recall” OR KW “consumer attitude*” OR KW “consumer engagement” OR KW “click-through-rate” OR KW “customer engagement” OR KW “customer recall” OR KW “customer attitude*” OR KW “comprehension” OR KW “Disclosure*” OR KW “Required disclosure language” OR KW “required disclosure” OR KW disclaimer Limiters - Publication Year: 1997-2020; Publication Type: Peer Reviewed Journal; English

**Total**

S4 S1 AND S2 AND S3 Limiters - Publication Year: 1997-2020; Publication Type: Peer Reviewed Journal; English

**Business Source Corporate (review articles)**

**January 1, 1997-February 17, 2021**

**Limit to academic journals and review articles**

S1 SU “Computers in advertising” OR SU “Internet Advertising” OR SU “Interactive marketing” OR SU “Click through rate” OR SU “Internet banner advertising” OR SU “Banner advertisements” OR SU “Banner advertising “ OR SU “Internet pop-up advertising” OR SU “Internet marketing” OR SU “Interactive multimedia” OR KW “Interactive advertising” OR KW “Information presentation features” OR KW “Digital marketing” OR KW “Banner advertising” Limiters - Published Date: 19970101-20201231; Publication Type: Academic Journal 2,980

S2 (KW “literature review*” OR KW review OR KW “systematic review*” OR KW “narrative review*” OR TI review) NOT (TI “online review*” OR KW “online review*” OR TI “consumer review*” OR TI “product review*” OR TI “user review*” OR TI “ratings reviews” OR TI “conference review” OR KW “online product review*” OR KW “vacation review*” OR KW “product review*” OR KW “customer review*” OR DE “CONSUMERS' reviews” OR DE “ONLINE comments” OR DE Product Reviews OR DE “PROFESSIONAL peer review”) Limiters - Published Date: 19970101-20201231; Publication Type: Academic Journal 22,948

S3 S1 AND S2 AND ZL “english” Limiters - Published Date: 19970101-20201231; Publication Type: Academic Journal

**Business Source Corporate (primary articles)**

**January 1, 1997-February 17, 2021**

**Limit to academic journals**

S1 SU “Advertising effectiveness” OR SU “Advertising campaigns” OR SU “Computers in advertising” OR SU “Internet Advertising” OR SU “Interactive marketing” OR SU “In-stream advertising” OR SU “Click through rate” OR SU “Internet banner advertising” OR SU “Banner advertisements” OR SU “Banner advertising “ OR SU “Internet pop-up advertising” OR SU “Internet marketing” OR SU “Interactive multimedia” OR KW “Interactive advertising” OR KW “Information presentation features” OR KW “Digital marketing” OR KW “Banner advertising” Limiters - Published Date: 19970101-20201231; Publication Type: Academic Journal 4,289

S2 SU “False advertising” OR SU “Selective dissemination of information” OR SU “Disclosure” OR SU “Disclaimers” OR SU “Inadequate disclosure” OR SU “Disclosure of information” OR SU “Financial disclosure” OR SU “Investor protection” OR SU “Consumer protection” OR SU “Risk perception” OR SU “Risk assessment” OR SU “Psychology” OR SU “Consumer attitudes” OR SU “Consumer behavior” OR SU “Consumer research” OR SU “Consumer preferences” OR SU “Consumer psychology” OR SU “Consumer education” OR SU “Information-seeking behavior” OR SU “RECOGNITION (Psychology)” OR KW “Interactive disclosures” OR KW “Paradigm” OR KW “Framework” Limiters - Published Date: 19970101-20201231; Publication Type: Academic Journal 127,985

S3 S1 AND S2 AND ZL “english” Limiters - Published Date: 19970101-20201231; Publication Type: Academic Journal

**SCOPUS**

**January 1, 2000 to February 17, 2021**

KEY(interactive advertising) OR KEY(digital advertising) OR KEY(internet advertising) OR KEY(Social Media Advertising)

Limit to SUBJAREA(BUSI)
