# Supplementary material for: The impact of interactive advertising on consumer engagement, recall, and understanding: A scoping systematic review for informing regulatory science
Source: PLoS One. 2022 Feb 3;17(2):e0263339. doi: 10.1371/journal.pone.0263339 (PMC8812936; doi:10.1371/journal.pone.0263339)
Supplement: S2 Appendix — (DOCX) [file pone.0263339.s003.docx]

## S2 Appendix. Study selection criteria

|  | Inclusion | Exclusion |
| --- | --- | --- |
| Population | All RQs: Studies among persons of any age in the general public who are characterized as being a potential consumer target for interactive advertising. | All RQs: Studies conducted among persons explicitly selected because of specific occupation or professional expertise (e.g., health care professionals, food or beverage distributors, financial advisors). |
| Exposure/  Intervention | All RQs: Exposure to interactive advertisements. We define interactive advertising as the promotion of a product, service, or idea using various features or tools that provide the opportunity for persons to interact directly with the ad and potentially influence/inform the remaining sequence, appearance, or content to be presented about the product, service, or idea. Differs from traditional media in that it involves an element of dynamic process, reciprocity, and bidirectional feedback/communication between the ad and the person viewing the ad. Includes banner or other online advertisements with embedded links, features such as quizzes or brief questionnaires, virtual reality, games, text message ads with embedded links, mobile location-based advertising, QR codes, requests to like, share, or comment on promoted content, and hovering over an element to show additional content.    RQ 2: Exposure to interactive advertising in naturalistic or real-world contexts | All RQs:  Exposure to:   - Traditional print or broadcast advertisements - Internet and online ads that do not include interactive elements (e.g., interstitial ads, pop-up ads, or banner ads that display information but do not offer any opportunity to interact or control navigation within the ad) - Interactive ads primarily using influence marketing through social media. - Digital ads that vary in text or graphic content or tone, placement, frequency of exposure, degree of personalization based on data already available on consumer - Websites in general - Ads designed to recruit participants for research studies - Interactivity in media other than advertisements (e.g., menus)     RQ 2: Exposure to interactive ads in a laboratory context (e.g., cognitive testing, market research, usability testing) |
| Comparator Interventions | RQ 1 and RQ 2: Any comparator or no comparator.    RQ 3: Alternative versions of advertisements with interactive elements that vary with respect to the type or level of interactivity.  RQ 4:   - Print ads - Broadcast ads - Online/internet ads without interactive elements | RQ 1 and RQ 2: No exclusions  RQ 3 and RQ 4: No comparator (i.e., no within-group or between-group comparisons reported); ads that compare various images or text content, or website placement or level of haptics, but do not compare different interactive features were not eligible. |
| Outcomes | RQ 1: Measures of consumer engagement.    RQ 2: Objective measures of consumer engagement with ad (e.g., time spent viewing, content navigation, click-through rates, page views, shares, likes, leaving comments).    RQs 3 and 4: Consumer engagement, recall, awareness, and comprehension of product claims, risk disclosures, or both. | RQs 1, 2, 3, 4: Any outcomes not specified as included.  RQs 2, 3, 4: Subjective measures of engagement (e.g., data collected via survey or interview that is based on respondent recall or that asks respondents about their intent to engage). |
| Study Design | All RQs: Research studies with primary data collection and analysis or secondary analysis of existing data focused on evaluating eligible interventions as specified above. | - Content analyses of websites, social media pages, or other digital platforms - Commentaries - Editorials - Narrative and systematic reviews are not eligible for inclusion but were searched for potentially relevant studies.     RQs 3 and 4: Qualitative research designs. |
| Other | All RQs: Published in English in 1997 or later; conducted in countries designated as *very highly developed* per the United Nations Human Development Index* This refers to the population evaluated by studies, not the author’s affiliation or location.  Publication in peer-reviewed journals. | All RQs: Published in languages other than English or prior to 1997; conducted in countries designated as less than *very highly developed* per the United Nations Human Development Index.  Dissertations, studies, or articles published in forums other than peer-reviewed journals. |

* Andorra, Argentina, Australia, Austria, Bahamas, Bahrain, Barbados, Belarus, Belgium, Brunei Darussalam, Bulgaria, Canada, Chile, Costa Rica, Croatia, Cyprus, Czechia, Denmark, Estonia, Finland, France, Georgia, Germany, Greece, Hong Kong China (SAR), Hungary, Iceland, Ireland, Israel, Italy, Japan, Kazakhstan, Korea (Republic of), Kuwait, Latvia, Liechtenstein, Lithuania, Luxembourg, Malaysia, Malta, Mauritius, Montenegro, Netherlands, New Zealand, Norway, Oman, Palau, Panama, Poland, Portugal, Qatar, Romania, Russian Federation, Saudi Arabia, Serbia, Singapore, Slovakia, Slovenia, Spain, Sweden, Switzerland, Turkey, United Arab Emirates, United Kingdom, United States, Uruguay

Abbreviations: QR, quick response; RQ, research question
