# Supplementary material for: The impact of interactive advertising on consumer engagement, recall, and understanding: A scoping systematic review for informing regulatory science
Source: PLoS One. 2022 Feb 3;17(2):e0263339. doi: 10.1371/journal.pone.0263339 (PMC8812936; doi:10.1371/journal.pone.0263339)
Supplement: S3 Appendix — (DOCX) [file pone.0263339.s004.docx]

## S3 Appendix. List of excluded studies

Alalwan, Ali Abdallah (2018), "Investigating the Impact of Social Media Advertising Features on Customer Purchase Intention," *International Journal of Information Management*, 42, 65-77.

Arroyo-Cañada, F. J., and J. Gil-Lafuente (2012), "Considerations of Interactive Digital Television as Advertising Media," *Journal of Promotion Management*, 18(3), 306-318.

Baron, Shawn D., Caryn Brouwer, and Amaya Garbayo (2014), "A Model for Delivering Branding Value through High-Impact Digital Advertising: How High-Impact Digital Media Created a Stronger Connection to Kellogg's Special K®," *Journal of Advertising Research*, 54(3), 286-291.

Belanche, D., C. Flavián, and A. Pérez-Rueda (2017), "Understanding Interactive Online Advertising: Congruence and Product Involvement in Highly and Lowly Arousing, Skippable Video Ads," *Journal of Interactive Marketing*, 37, 75-88.

Bolton, Ruth, and Shruti Saxena-Iyer (2009), "Interactive Services: A Framework, Synthesis and Research Directions," *Journal of Interactive Marketing*, 23(1), 91-104.

Brasel, S. Adam (2011), "Nonconscious Drivers of Visual Attention in Interactive Media Environments," *Journal of Brand Management*, 18(7), 473−482.

Buchanan, L., B. Kelly, H. Yeatman, and K. Kariippanon (2018), "The Effects of Digital Marketing of Unhealthy Commodities on Young People: A Systematic Review," *Nutrients*, 10(2).

Buchanan, L., H. Yeatman, B. Kelly, and K. Kariippanon (2018), "A Thematic Content Analysis of How Marketers Promote Energy Drinks on Digital Platforms to Young Australians," *Australian and New Zealand Journal of Public Health*, 42(6), 530-531.

Bucy, Erik P. (2004), "The Debate," *Information Society*, 20(5), 371-371.

Bucy, Erik P. (2004), "Interactivity in Society: Locating an Elusive Concept," *Information Society*, 20(5), 373-383.

Burns, Kelli S., and Richard J. Lutz (2006), "The Function of Format: Consumer Responses to Six on-Line Advertising Formats," *Journal of Advertising*, 35(1), 53-63.

Cairns, Georgina (2013), "Evolutions in Food Marketing, Quantifying the Impact, and Policy Implications," *Appetite*, 62, 194-197.

Calder, Bobby J., Edward C. Malthouse, and Ute Schaedel (2009), "An Experimental Study of the Relationship between Online Engagement and Advertising Effectiveness," *Journal of Interactive Marketing*, 23(4), 321-331.

Cassioli, F. (2019), "Contents, Animation or Interactivity: Neurophysiological Correlates in App Advertising," *Neuropsychological Trends*(26), 83-91.

Cauberghe, Verolien, and Patrick De Pelsmacker (2008), "The Advertising Impact of an Interactive TV Program on the Recall of an Embedded Commercial," *Journal of Advertising Research*, 48(3), 352-362.

Cauberghe, Verolien, and Patrick De Pelsmacker (2008), "The Impact of Banners on Digital Television: The Role of Program Interactivity and Product Involvement," *CyberPsychology & Behavior*, 11(1), 91-94.

Cavallo, D., R. Lim, K. Ishler, M. Pagano, R. Perovsek, E. Albert, S. Koopman Gonzalez, E. Trapl, and S. Flocke (2020), "Effectiveness of Social Media Approaches to Recruiting Young Adult Cigarillo Smokers: Cross-Sectional Study," *Journal of Medical Internet Research*, 22(7), e12619.

Chandon, Jean Louis, Mohamed Saber Chtourou, and David R. Fortin (2003), "Effects of Configuration and Exposure Levels on Responses to Web Advertisements," *Journal of Advertising Research*, 43(2), 217-229.

Chatterjee, Patrali, Donna L. Hoffman, and Thomas P. Novak (2003), "Modeling the Clickstream: Implications for Web-Based Advertising Efforts," *Marketing Science*, 22(4), 520-541.

Chen, Qimei, David A. Griffith, and Fuyuan Shen (2005), "The Effects of Interactivity on Cross-Channel Communication Effectiveness," *Journal of Interactive Advertising*, 5(2), N.PAG.

Cheung, Christy M. K., Gloria W. W. Chan, and Moez Limayem (2005), "A Critical Review of Online Consumer Behavior: Empirical Research," *Journal of Electronic Commerce in Organizations*, 3(4), 1-19.

Collins, L., A. M. Glasser, H. Abudayyeh, J. L. Pearson, and A. C. Villanti (2019), "E-Cigarette Marketing and Communication: How E-Cigarette Companies Market E-Cigarettes and the Public Engages with E-Cigarette Information," *Nicotine & Tobacco Research*, 21(1), 14-24.

Cook, Don Lloyd, and Eloise Coupey (1998), "Consumer Behavior and Unresolved Regulatory Issues in Electronic Marketing," *Journal of Business Research*, 41(3), 231−238.

Coyle, James R., and Esther Thorson (2001), "The Effects of Progressive Levels of Interactivity and Vividness in Web Marketing Sites," *Journal of Advertising*, 30(3), 65-77.

Cugelman, B., M. Thelwall, and P. Dawes (2011), "Online Interventions for Social Marketing Health Behavior Change Campaigns: A Meta-Analysis of Psychological Architectures and Adherence Factors," *Journal of Medical Internet Research*, 13(1), e17.

Danaher, Peter J., and Guy W. Mullarkey (2003), "Factors Affecting Online Advertising Recall: A Study of Students," *Journal of Advertising Research*, 43(3), 252-267.

Daugherty, Terry, Vanja Djuric, Hairong Li, and John Leckenby (2017), "Establishing a Paradigm: A Systematic Analysis of Interactive Advertising Research," *Journal of Interactive Advertising*, 17(1), 65−78.

Davis, K. C., P. R. Shafer, R. Rodes, A. Kim, H. Hansen, D. Patel, C. Coln, and D. Beistle (2016), "Does Digital Video Advertising Increase Population-Level Reach of Multimedia Campaigns? Evidence from the 2013 Tips from Former Smokers Campaign," *Journal of Medical Internet Research*, 18(9), e235.

Davis, Robert, and Laszlo Sajtos (2008), "Measuring Consumer Interactivity in Response to Campaigns Coupling Mobile and Television Media," *Journal of Advertising Research*, 48(3), 375-391.

Dennis, Charles, Bill Merrilees, Chanaka Jayawardhena, and Len Tiu Wright (2009), "E-Consumer Behaviour," *European Journal of Marketing*, 43(9-10), 1121-1139.

Derrick, J. L., R. K. Eliseo-Arras, C. Hanny, M. Britton, and S. Haddad (2017), "Comparison of Internet and Mailing Methods to Recruit Couples into Research on Unaided Smoking Cessation," *Addictive Behaviors*, 75, 12-16.

Diao, Fangfang, and S. Shyam Sundar (2004), "Orienting Response and Memory for Web Advertisements: Exploring Effects of Pop-up Window and Animation," *Communication Research*, 31(5), 537-567.

Dodoo, Naa Amponsah, and Linwan Wu (2021), "Regulatory Focus and Choice: The Impact of Control Perceptions on Advertising Effectiveness," *Journal of Marketing Communications*, 27(1), 69-92.

Dwivedi, Yogesh K., Kawaljeet Kaur Kapoor, and Hsin Chen (2015), "Social Media Marketing and Advertising," *Marketing Review*, 15(3), 289-309.

Florenthal, Bela, and Aviv Shoham (2010), "Four-Mode Channel Interactivity Concept and Channel Preferences," *Journal of Services Marketing*, 24(1), 29−41.

Fortin, David R., and Ruby Roy Dholakia (2005), "Interactivity and Vividness Effects on Social Presence and Involvement with a Web-Based Advertisement," *Journal of Business Research*, 58(3), 387−396.

Fulgoni, Gian M. (2016), "In the Digital World, Not Everything That Can Be Measured Matters," *Journal of Advertising Research*, 56(1), 9−13.

Gallagher, Katherine, K. Dale Foster, and Jeffrey Parsons (2001), "The Medium Is Not the Message: Advertising Effectiveness and Content Evaluation in Print and on the Web," *Journal of Advertising Research*, 41(4), 57-70.

Gao, Qin, Pei-Luen Patrick Rau, and Gavriel Salvendy (2009), "Perception of Interactivity: Affects of Four Key Variables in Mobile Advertising," *International Journal of Human-Computer Interaction*, 25(6), 479-505.

Gao, Qin, Pei-Luen Patrick Rau, and Gavriel Salvendy (2010), "Measuring Perceived Interactivity of Mobile Advertisements," *Behaviour & Information Technology*, 29(1), 35−44.

Goldfarb, Avi, and Catherine Tucker (2011), "Online Display Advertising: Targeting and Obtrusiveness," *Marketing Science*, 30(3), 389-404.

Griffith, David A., and Qimei Chen (2004), "The Influence of Virtual Direct Experience (Vde) on on-Line Ad Message Effectiveness," *Journal of Advertising*, 33(1), 55-68.

Hair, E., L. Pitzer, M. Bennett, M. Halenar, J. Rath, J. Cantrell, N. Dorrler, E. Asche, and D. Vallone (2017), "Harnessing Youth and Young Adult Culture: Improving the Reach and Engagement of the Truth® Campaign," *Journal of Health Communication*, 22(7), 568-575.

Häubl, Gerald, and Valerie Trifts (2000), "Consumer Decision Making in Online Shopping Environments: The Effects of Interactive Decision Aids," *Marketing Science*, 19(1), 4.

Hu, Xiaohan, and Kevin Wise (2020), "Perceived Control or Haptic Sensation? Exploring the Effect of Image Interactivity on Consumer Responses to Online Product Displays," *Journal of Interactive Advertising*, 20(1), 60-75.

Huang, Jinsong, Song Su, Liuning Zhou, and Xi Liu (2013), "Attitude toward the Viral Ad: Expanding Traditional Advertising Models to Interactive Advertising," *Journal of Interactive Marketing*, 27(1), 36-46.

Jin, C. H., and J. Villegas (2007), "Consumer Responses to Advertising on the Internet: The Effect of Individual Difference on Ambivalence and Avoidance," *Cyberpsychology & Behavior: the Impact of the Internet, Multimedia and Virtual Reality on Behavior and Society*, 10(2), 258-266.

Jin, Seunga Venus, and Joe Phua (2015), "The Moderating Effect of Computer Users’ Autotelic Need for Touch on Brand Trust, Perceived Brand Excitement, and Brand Placement Awareness in Haptic Games and in-Game Advertising (Iga)," *Computers in Human Behavior*, 43, 58-67.

Jung, J. M., H. C. Hui, K. S. Min, and D. Martin (2014), "Does Telic/Paratelic User Mode Matter on the Effectiveness of Interactive Internet Advertising? A Reversal Theory Perspective," *Journal of Business Research*, 67(6), 1303-1309.

Jung, Jae Min, Kyeong Sam Min, and James J. Kellaris (2011), "The Games People Play: How the Entertainment Value of Online Ads Helps or Harms Persuasion," *Psychology & Marketing*, 28(7), 661-681.

Kang, Hyo Jeong, Jung-hye Shin, and Kevin Ponto (2020), "How 3d Virtual Reality Stores Can Shape Consumer Purchase Decisions: The Roles of Informativeness and Playfulness," *Journal of Interactive Marketing*, 49, 70-85.

Kapoor, Kawaljeet Kaur, Yogesh K. Dwivedi, and Niall C. Piercy (2016), "Pay-Per-Click Advertising: A Literature Review," *Marketing Review*, 16(2), 183-202.

Khim-Yong, Goh, and Jerry Wenjie Ping (2014), "Engaging Consumers with Advergames: An Experimental Evaluation of Interactivity, Fit and Expectancy," *Journal of the Association for Information Systems*, 15(7), 388-421.

Kilger, Max, and Ellen Romer (2007), "Do Measures of Media Engagement Correlate with Product Purchase Likelihood?," *Journal of Advertising Research*, 47(3), 313-325.

Knoll, Johannes (2016), "Advertising in Social Media: A Review of Empirical Evidence," *International Journal of Advertising*, 35(2), 266-300.

Lee, D., K. Hosanagar, and H. S. Nair (2018), "Advertising Content and Consumer Engagement on Social Media: Evidence from Facebook," *Management Science*, 64(11), 5105-5131.

Lee, Joonghwa, Hyojung Park, and Kevin Wise (2014), "Brand Interactivity and Its Effects on the Outcomes of Advergame Play," *New Media & Society*, 16(8), 1268-1286.

Levy, Shalom, and I. D. Nebenzahl (2008), "The Influence of Product Involvement on Consumers' Interactive Processes in Interactive Television," *Marketing Letters*, 19(1), 65−77.

Liu, Yuping, and L. J. Shrum (2002), "What Is Interactivity and Is It Always Such a Good Thing? Implications of Definition, Person, and Situation for the Influence of Interactivity on Advertising Effectiveness," *Journal of Advertising*, 31(4), 53-64.

Liu, Yuping, and L. J. Shrum (2009), "A Dual-Process Model of Interactivity Effects," *Journal of Advertising*, 38(2), 53-68.

Lobstein, T., J. Landon, N. Thornton, and D. Jernigan (2017), "The Commercial Use of Digital Media to Market Alcohol Products: A Narrative Review," *Addiction*, 112 Suppl 1, 21-27.

McFarland, R. G., S. V. Dalsem, and C. Sheu (2004), "Developing Interactive Advertising Strategies on the Internet as a Function of Consumer Knowledge, Ability, and Involvement," *International Journal of Internet Marketing and Advertising*, 1(3), 300−315.

McKeever, R. (2014), "Thinking Outside the Medicine Cabinet: A Comparative Content Analysis of Direct-to-Consumer Advertisements for Prescription Drug Treatments," *Health Marketing Quarterly*, 31(4), 353-369.

McMillan, Sally J., and Jang-Sun Hwang (2002), "Measures of Perceived Interactivity: An Exploration of the Role of Direction of Communication, User Control, and Time in Shaping Perceptions of Interactivity," *Journal of Advertising*, 31(3), 29−42.

Mollen, Anne, and Hugh Wilson (2010), "Engagement, Telepresence and Interactivity in Online Consumer Experience: Reconciling Scholastic and Managerial Perspectives," *Journal of Business Research*, 63(9-10), 919−925.

Montgomery, Alan L., and Michael D. Smith (2009), "Prospects for Personalization on the Internet," *Journal of Interactive Marketing*, 23(2), 130-137.

Montgomery, K. C., and J. Chester (2009), "Interactive Food and Beverage Marketing: Targeting Adolescents in the Digital Age," *Journal of Adolescent Health*, 45(3 Suppl), S18-29.

Oh, Jeeyun, Hayoung Sally Lim, and Angel Hsing-Chi Hwang (2020), "How Interactive Storytelling Persuades: The Mediating Role of Website Contingency and Narrative Transportation," *Journal of Broadcasting & Electronic Media*, 64(5), 714-735.

Ratchford, Brian T. (2015), "Some Directions for Research in Interactive Marketing," *Journal of Interactive Marketing*, 29, v-vii.

Redondo, Ignacio (2012), "The Effectiveness of Casual Advergames on Adolescents' Brand Attitudes," *European Journal of Marketing*, 46(11/12), 1671-1688.

Rishika, Rishika, Ashish Kumar, Ramkumar Janakiraman, and Ram Bezawada (2013), "The Effect of Customers’ Social Media Participation on Customer Visit Frequency and Profitability: An Empirical Investigation," *Information Systems Research*, 24(1), 108-127.

Robertson, A., D. T. Morse, K. Hood, and C. Walker (2017), "Measuring Alcohol Marketing Engagement: The Development and Psychometric Properties of the Alcohol Marketing Engagement Scale," *Journal of Applied Measurement*, 18(1), 87-99.

Seo, Yuri, Jungkeun Kim, Yung Kyun Choi, and Xiaozhu Li (2019), "In "Likes" We Trust: Likes, Disclosures and Firm-Serving Motives on Social Media," *European Journal of Marketing*, 53(10), 2173-2192.

Shankar, Venkatesh, and Edward C. Malthouse (2009), "A Peek into the Future of Interactive Marketing," *Journal of Interactive Marketing*, 23(1), 1-3.

Sicilia, Maria, Salvador Ruiz, and Jose L. Munuera (2005), "Effects of Interactivity in a Web Site: The Moderating Effect of Need for Cognition," *Journal of Advertising*, 34(3), 31-45.

Sohn, Dongyoung, Cunhyeong Ci, and Byung-Kwan Lee (2007), "The Moderating Effects of Expectation on the Patterns of the Interactivity-Attitude Relationship," *Journal of Advertising*, 36(3), 109-119.

Stewart, David W., and Paul A. Pavlou (2002), "From Consumer Response to Active Consumer: Measuring the Effectiveness of Interactive Media," *Journal of the Academy of Marketing Science*, 30(4), 376-396.

Sullivan, H. W., A. C. O'Donoghue, D. J. Rupert, J. F. Willoughby, and K. J. Aikin (2017), "Placement and Format of Risk Information on Direct-to-Consumer Prescription Drug Websites," *Journal of Health Communication*, 22(2), 171-181.

Sundar, S. Shyam (2004), "Theorizing Interactivity's Effects," *Information Society*, 20(5), 385−389.

Sundar, S. Shyam, and Jinhee Kim (2005), "Interactivity and Persuasion: Influencing Attitudes with Information and Involvement," *Journal of Interactive Advertising*, 5(2), N.PAG.

Tan, B. J., M. Brown, and N. Pope (2019), "The Role of Respect in the Effects of Perceived Ad Interactivity and Intrusiveness on Brand and Site," *Journal of Marketing Communications*, 25(3), 288−306.

Tattan-Birch, H., S. E. Jackson, C. Ide, L. Bauld, and L. Shahab (2020), "Evaluation of the Impact of a Regional Educational Advertising Campaign on Harm Perceptions of E-Cigarettes, Prevalence of E-Cigarette Use, and Quit Attempts among Smokers," *Nicotine & Tobacco Research*, 22(7), 1148-1154.

Taylor, Charles R. (2009), "The Six Principles of Digital Advertising," *International Journal of Advertising*, 28(3), 411-418.

Tsai, Wan-Hsiu Sunny, Shiyun Chloe Tian, Ching-Hua Chuan, and Cong Li (2020), "Inspection or Play? A Study of How Augmented Reality Technology Can Be Utilized in Advertising," *Journal of Interactive Advertising*, 20(3), 244-257.

Uzunoğlu, Ebru, and Burcu Öksüz (2014), "New Opportunities in Social Media for Ad-Restricted Alcohol Products: The Case of ‘Yeni Rakı’," *Journal of Marketing Communications*, 20(4), 270-290.

Van den Broeck, E., B. Zarouali, and K. Poels (2019), "Chatbot Advertising Effectiveness: When Does the Message Get Through?," *Computers in Human Behavior*, 98, 150-157.

van Noort, Guda, Marjolijn L. Antheunis, and Peeter W. J. Verlegh (2014), "Enhancing the Effects of Social Network Site Marketing Campaigns: If You Want Consumers to Like You, Ask Them About Themselves," *International Journal of Advertising: The Quarterly Review of Marketing Communications*, 33(2), 135-152.

van Noort, Guda, Hilde A. M. Voorveld, and Eva A. van Reijmersdal (2012), "Interactivity in Brand Web Sites: Cognitive, Affective, and Behavioral Responses Explained by Consumers' Online Flow Experience," *Journal of Interactive Marketing*, 26(4), 223-234.

van Reijmersdal, Eva A., Jeroen Jansz, Oscar Peters, and Guda van Noort (2010), "The Effects of Interactive Brand Placements in Online Games on Children’s Cognitive, Affective, and Conative Brand Responses," *Computers in Human Behavior*, 26(6), 1787-1794.

van Reijmersdal, Eva A., Esther Rozendaal, and Moniek Buijzen (2012), "Effects of Prominence, Involvement, and Persuasion Knowledge on Children's Cognitive and Affective Responses to Advergames," *Journal of Interactive Marketing*, 26(1), 34-42.

Vanwesenbeeck, I., L. Hudders, and K. Ponnet (2020), "Understanding the Youtube Generation: How Preschoolers Process Television and Youtube Advertising," *Cyberpsychol Behav Soc Netw*, 23(6), 426-432.

Varadarajan, Rajan, Raji Srinivasan, Gautham Gopal Vadakkepatt, Manjit S. Yadav, Paul A. Pavlou, Sandeep Krishnamurthy, and Tom Krause (2010), "Interactive Technologies and Retailing Strategy: A Review, Conceptual Framework and Future Research Directions," *Journal of Interactive Marketing (Elsevier)*, 24(2), 96-110.

Vashisht, Devika, and Abhishek Chauhan (2017), "Effect of Game-Interactivity and Congruence on Presence and Brand Attitude," *Marketing Intelligence & Planning*, 35(6), 789-804.

Vashisht, Devika, and Sreejesh S. Pillai (2017), "Are You Able to Recall the Brand? The Impact of Brand Prominence, Game Involvement and Persuasion Knowledge in Online – Advergames," *Journal of Product & Brand Management*, 26(4), 402-414.

Voorveld, Hilde A. M., Peter C. Neijens, and Edith G. Smit (2011), "The Relation between Actual and Perceived Interactivity: What Makes the Web Sites of Top Global Brands Truly Interactive?," *Journal of Advertising*, 40(2), 77-92.

Wang, Weiquan, and Izak Benbasat (2009), "Interactive Decision Aids for Consumer Decision Making in E-Commerce: The Influence of Perceived Strategy Restrictiveness," *MIS Quarterly*, 33(2), 293-320.

Wise, Kevin, Paul D. Bolls, Hyo Kim, Arun Venkataraman, and Ryan Meyer (2008), "Enjoyment of Advergames and Brand Attitudes: The Impact of Thematic Relevance," *Journal of Interactive Advertising*, 9(1), 14-36.

Wu, G. (2006), "Conceptualizing and Measuring the Perceived Interactivity of Websites," *Journal of Current Issues and Research in Advertising*, 28(1), 87−104.

Yaoyuneyong, Gallayanee, Jamye Foster, Erik Johnson, and David Johnson (2016), "Augmented Reality Marketing: Consumer Preferences and Attitudes toward Hypermedia Print Ads," *Journal of Interactive Advertising*, 16(1), 16-30.

Yim, Mark Yi-Cheon, Shu-Chuan Chu, and Paul L. Sauer (2017), "Is Augmented Reality Technology an Effective Tool for E-Commerce? An Interactivity and Vividness Perspective," *Journal of Interactive Marketing (Elsevier)*, 39, 89-103.

Yoo, Chul Woo, Yong Jin Kim, and G. Lawrence Sanders (2015), "The Impact of Interactivity of Electronic Word of Mouth Systems and E-Quality on Decision Support in the Context of the E-Marketplace," *Information & Management*, 52(4), 496-505.

Yoon, Doyle, and Seounmi Youn (2016), "Brand Experience on the Website: Its Mediating Role between Perceived Interactivity and Relationship Quality," *Journal of Interactive Advertising*, 16(1), 1-15.

Zhao, J., and J. Wang (2020), "Health Advertising on Short-Video Social Media: A Study on User Attitudes Based on the Extended Technology Acceptance Model," *International Journal of Environmental Research and Public Health*, 17(5).

Zhenhui, Jiang, and Izak Benbasat (2007), "Investigating the Influence of the Functional Mechanisms of Online Product Presentations," *Information Systems Research*, 18(4), 454-470.
