# Supplementary material for: The impact of interactive advertising on consumer engagement, recall, and understanding: A scoping systematic review for informing regulatory science
Source: PLoS One. 2022 Feb 3;17(2):e0263339. doi: 10.1371/journal.pone.0263339 (PMC8812936; doi:10.1371/journal.pone.0263339)
Supplement: S4 Appendix — (DOCX) [file pone.0263339.s005.docx]

## S4 Appendix. Characteristics and results from studies included in research questions 1 through 4

**Table S4-1. Description of Included Studies**

| **Author;**  **Year** | **Country** | **Brief study description** | **Applicable research questions** | **Type of product/service advertised** |
| --- | --- | --- | --- | --- |
| Barreto[50];  2013 | US | Experimental study with university students (N = 20) using eye-tracking to measure participants’ fixation time on Facebook banner ads. | RQ 1 | Consumer product (unregulated) |
| Bellman et al.[49]; 2014 | Australia | Experimental study among participants (N = 233) comparing the effectiveness of PC advergames, TV commercials, and interactive commercials with advergames exposure on brand attitude and day-after recall of ad design and content. | RQ 4 | Consumer product (unregulated) |
| Bellman, Schweda, and Varan[52]; 2010 | Australia | 2 (interactivity) X 3 (number of exposures) X 2 (brand familiarity) factorial experiment with interactive television commercials among Australian audience panel members (N = 273) to identify the minimum effective frequency of interactive TV ads as assessed by direct response to ad. | RQ 1 | Consumer product (unregulated) and regulated product or service (car insurance) |
| Bezjian-Avery, Calder, and Iacobucci[7]; 1998 | US | Experimental study comparing consumer (N = 96) attitudes toward interactive vs. linear presentation of dessert and beverage ads. Ads were static, but experimental group participants controlled ad presentation order. Measured recall, brand attitude, and purchase intention via survey, as well as view duration per ad. | RQ 1 | Consumer product (unregulated) |
| Birnbaum et al.[29]; 2017 | US | Observational study evaluating the effect of Google AdWords ads directed toward help-seeking individuals with early-stage psychotic disorders in New York City metro area (N = NR) on website engagement. | RQ 1  RQ 2 | Health/health behavior |
| Cauberghe and De Pelsmacker[51]; 2010 | Belgium | 2 (amount of information) X 3 (interactivity) factorial experiment among market research participants (N = 282) exploring the effects of differing levels of ad interactivity and information within an interactive TV ad on user brand attitudes, recall, and time spent in the dedicated ad location. | RQ 1  RQ 3 | consumer service (unregulated) |
| Cho[33]; 2003 | NR | 2 (product) X 3 (presentation order) factorial experiment of website banner ad exposure with a nonrandom sample (N = 756) recruited from education- and internet-related listservs evaluating click-through rates, level of involvement, and attitudes. | RQ 1 | consumer product (unregulated) |
| Cho, Lee, and Tharp[35]; 2001 | NR | Experimental study of participants recruited from education-, internet- and marketing-related listservs (N = 215) exposed to 4 banner ads for fictional brands and services to determine click-through rates and whether the level of ad exposure affects ad recall and awareness. | RQ 1 | Consumer products and service (unregulated) and regulated service (financial) |
| Chongyu et al.[54]; 2020 | NR | Observational study of daily Google AdWords data from 13 advertisers using paid search advertising campaigns to investigate differences in consumer (N = NR) click-through behavior and differences between platforms (i.e., desktop, smartphone, tablet). | RQ 2 | Consumer product and service (unregulated) |
| Chung and Ahn [32]; 2007 | US | 2 (personality difference) X 4 (website structure) factorial experiment among undergraduate students (N = 181) evaluating the most effective web ad structure on memory, attitude, and behavioral intentions. | RQ 3 | Consumer product (unregulated) |
| Chung and Zhao[36]; 2004 | US | 2 (personal motivation) X 3 (number of hyperlinks) factorial experiment among university students (N = 180) with exposure to a fictitious product (camera) website to evaluate the effects on website surfing behaviors and advertising effectiveness. | RQ 1  RQ 3 | Consumer product (unregulated) |
| Daems, De Pelsmacker, and Moons[43]; 2019 | Belgium | 2 (integration) X 2 (brand interactivity) factorial experiment among children in 7^th^ or 8^th^ grade (N = 576) examining the effect of brand interactivity and integration in other content for a fictitious smartphone ad on brand memory, awareness of selling intent, critical processing, brand attitude, and personal information sharing. | RQ 3 | Consumer product (unregulated) |
| Graham et al.[30]; 2008 | US | Observational study comparing online advertising with traditional for recruiting individuals (N = NR) to smoking cessation treatment. Ads used creative messaging and directed viewers to the QuickNet website to register for cessation treatments. | RQ 1  RQ 2  RQ 4 | Health/health behavior |
| Horrell et al.[24]; 2019 | US | Observational study of user (N = 91,385) engagement with 5 weeklong Facebook advertising campaigns, each including 3 unique ads, all seeking to increase membership in an online community related to lung cancer. | RQ 1  RQ 2 | Health/health behavior |
| Ieva et al.[48]; 2018 | Italy | Experimental study comparing digital flyer delivered via email vs. traditional print flyer among a random sample of retail customers from an Italian supermarket’s loyalty card database (N = 9,902 randomized and included in purchase behavior analyses, 1,222 of which participated in follow-up telephone survey assessing recall). | RQ 4 | Consumer product (unregulated) |
| Kim et al.[28]; 2016 | US | Observational study of web users (N = 30,638) who were exposed or not exposed to ads for the Tips From Former Smokers 2012 campaign to assess whether exposure was associated with online searches or visits to campaign-related websites. | RQ 1  RQ 2 | Health/health behavior |
| Levy et al.[38, 55]; 2006, 2008 | Israel | 2 (TV program) X 4 (sequence of paired commercials for 4 different types of products) factorial experiement among univesity students (N = 243) assessing test ads in a factorial design assessing interactive behavior with ads. | RQ 1 | Consumer product and service (unregulated) |
| Macias[44]; 2003 | US | 2 (interactivity) X 2 (product) factorial experiment among undergraduate students (N = 153) to evaluate how the level of ad interactivity in product websites influences comprehension and attitudes. | RQ 3 | Consumer product (unregulated) |
| Moe[39]; 2006 | US | Experimental study of real-world users (N = 128 090) of an informational website about movies that assessed the impact of timing of popup ads offering subscription to a weekly email using click-through rates and page views. | RQ 1  RQ 2 | Consumer service (unregulated) |
| Munoz-Leiva, Hernández-Méndez, and Gómez-Carmona[26]; 2019 | Spain | Experimental study of banner ad effectiveness on Travel 2.0 websites. Social network users (N = 60) viewed 3 websites with an embedded ad. Study used eye-tracking and self-report survey as measures of ad effectiveness/recall. | RQ 1  RQ 3 | Consumer service (unregulated) |
| Noel and Babor[27]; 2018 | US | 2 (regulatory compliant) X 2 (user engagement) X 2 (user-generated comments) factorial experiment used to determine if user engagement values and user-generated comments displayed alongside alcohol ads on social networking sites can increase ad engagement and the desire to drink among adults aged 21 to 24 (N = 120). | RQ 1 | Regulated product or service |
| Platt et al.[31]; 2013 | US | Observational analysis of Facebook ad campaign targeted to Michigan residents aged 18 to 28 (N = ~2 million) to promote awareness of state biobank and evaluated with engagement measures. | RQ 2 | Health/health behavior |
| Polster et al.[34]; 2017 | US | Experimental analysis of whether important safety information is noticed and recalled on mocked-up websites for a fictitious asthma product among patients (N = 1,600). | RQ 3 | Regulated product or service |
| Rauwers, Voorveld, and Neijens[42]; 2018 | NL | Experimental study among volunteer readers of a women’s digital magazine (N = 98), some of whom were exposed to an Interactive advertisement (i.e., movie clip) to determine interactive feature use, ad exposure time, movie clip exposure time, app opening, attitudes about the ad, and perceived interactivity. | RQ 1 | Consumer product (unregulated) |
| Soneji et al.[25];  2019 | US | Observational study of adolescents enrolled in the nationally representative Population Assessment for Tobacco and Health study, comparing engagement with online tobacco marketing over time (2013–2014 [n = 13 651] compared with 2014–2015 [n = 12,172]). | RQ 1 | Regulated product or service |
| Trappey and Woodside[37]; 2005 | US and UK | Observational study analyzing findings from US and UK SMS-TV marketing campaigns (inbound and outbound), taken from existing database of consumer surveys and interviews (N varies by analysis). Outcomes include self-reported actions taken by consumers in response to SMS-TV marketing measured by survey or interview. | RQ 1 | Consumer product and service (unregulated) |
| Urban et al.[47];  2014 | Multiple | Experimental study among active consumers on CNET website (N = 116,168) who were randomized to test and control groups over the period of a month to evaluate performance of banner ad morphing and context matching on click-through rates. | RQ 1  RQ 2 | Consumer product (unregulated) |
| Yang[53]; 1997 | US | Experimental study among graduate students (N = 108) comparing the effectiveness of interactive and noninteractive ads for batteries and floppy disks by measuring average time spent in each ad type and brand and ad attitude. | RQ 1  RQ 3 | Consumer product (unregulated) |
| Yang and Shen[45]; 2018 | NR | Systematic review and meta-analysis of 63 experimental studies (N = 13,484) reporting on web interactivity (51% of studies were in an advertising context) | RQ 3 | NR |
| Yi, Jiang, and Benbasat[46]; 2015 | NR | Experimental study with students and staff from a public university (N = 170) presented with 1 of 3 different online formats (noninteractive video, full interaction, restricted interaction) for a fictitious cell phone brand/model to assess impact on engagement, enticement, and purchase intention. | RQ 1 | Consumer product (unregulated) |
| Yoo[40]; 2009 | US | Two experiments among undergraduates (N = 180 and N = 124) to evaluate conditions most conducive to incidental exposure to banner web ads and impact on implicit memory, click-through intention, and priming. | RQ 1 | Consumer product (unregulated) |
| Yoo[41]; 2011 | US | Experimental study with undergraduate students (N = 166) examining the effects of message framing, keyword insertion, and users’ level of product involvement on click-through behavior in response to 1 of 4 fictitious ads shown after searching a fictitious search engine using the term “discount.” | RQ 1 | Consumer product (unregulated) |

Abbreviations: CNET, computer network (an American media site); N, number; NL, The Netherlands; NR, not reported; PC, personal computer; RQ, research question; SMS-TV, short-message-service TV; TV, television; UK, United Kingdom; US, United States.

**Table S4-2. Methods and Measures Used to Assess Consumer Engagement (Research Question 1)**

| Author (year) | Objective | Population | Design/methods/measures/advertisement exposure | Other relevant RQs | |
| --- | --- | --- | --- | --- | --- |
| Barreto [50] | To determine whether Facebook users look at banner ads. | Undergraduate and graduate students in the US (N = 20) | Experimental study using participants’ actual Facebook account sites with timed exposure to a brand site of the participant’s choice and also to a Nike sports brand page; attention to banner ads measured with fixations and saccades using eye-tracking technology followed by a questionnaire about Facebook usage habits.  *Exposure:* Facebook social networking site, which contained participants’ personal Facebook accounts (“The Wall” + “News Feed”) and actual brand pages with embedded banner ads. All participants were asked to interact with their Facebook accounts as they would usually do for 2 minutes, next they were asked to look for any brand of their choice on the site and freely interact with it for 1.5 minutes, and then they were asked to interact with the Nike sports brand page for 1.5 minutes. | None | |
| Bellman, Schweda, and Varan [52] | To identify the minimum effective frequency for TV ads offering interactive response. | Australian audience panel (N = 273, representative of the general public). Sixty-six participants were excluded from analysis because of data issues. | Experimental design varying test TV ads by interactivity, repetition, and brand familiarity. Participants completed surveys immediately after viewing the program and again after 24 hours for unaided brand recall, brand-cued ad recognition, brand and ad attitudes, and purchase readiness. Cox regression was used to determine how many exposures an ad requires before a participant interacts with it.  *Exposure:* Participants watched 1 of 4 different hour-long US TV programs that each included 6 commercial ad breaks: 1 before and after and 4 during the show, and each break included 5 ads with the middle 3 positions serving as the test ads. Ads were for 5 product categories (car insurance [2 brands], nuts, salad dressing, soda, video camcorders). Participants were randomly assigned to test ads in a 2 (interactive vs. noninteractive between-subjects) X 3 (1, 3, or 5 exposures within-subjects) X 2 (brand familiarity vs. unfamiliarity within-subjects) factorial design. The interactive ads used the impulse response format, which is the closest iTV ad format to traditional direct response ads and has been used on the Wink platform in the United States. The interactive part included “call-to-action” banners superimposed over the normal TV ads and offered entry into a competition for a prize. | None | |
| Bezjian-Avery, Calder, and Iacobucci [7] | To compare consumer reactions to an interactive presentation of products vs. a linear presentation. | People of all ages (N = 96) recruited in a Midwest US restaurant | Participants viewed either an interactive or linear shopping website. Experimental group could choose what products to see; control group viewed all products in set linear order. All participants viewed all 8 ads. View duration recorded for each ad. A survey post-exposure measured recall, brand attitude, and purchase likelihood and whether participants were visual or verbal processors.  *Exposure:* Four ads for beverage and dessert products (product image plus ad text). Two were more “visual” ads, and 2 were more “verbal” ads. Participants viewed a shopping website with the 4 stimuli plus 4 filler ads. | None | |
| Birnbaum et al. [29] | To identify the benefits and challenges of using Google AdWords to engage youth with early-stage psychotic disorders and direct them toward treatment. | Individuals in the New York City metro area seeking help for early-stage psychotic disorders (N = NR) | Observational analysis of a 14-week long advertising campaign using Google AdWords to strategically place ads on Google search results pages directing users to a landing page for an early treatment program, cued by list of relevant search terms; Google Analytics was used to record website engagement and user demographic information.  *Exposure:* 154 unique ads were created, cued by more than 2,000 relevant search terms (containing words like “psychosis,” “hearing voices,” “mind control,” “bipolar disorder”) and organized across 15 thematic ad groups; each ad contained a headline (thematically related to each user’s search term), a description line (supporting information), and a call to action (encouraging users to click the ad, take a quiz, find support, for example). Unclear whether users were able to get more information or take a quiz within the ad itself or if they were immediately directed to the landing page for the early treatment program. | [RQ 2](#T2Birnbaumetal2017) | |
| Cauberghe et al. [51] | To investigate the effect of 2 telescopic ad characteristics on users’ time spent in the dedicated ad location, brand recall, and attitude toward brand in interactive TV scenarios. | Belgium residents from a market research agency randomly selected on the basis of age, gender, and education who were willing to participate (N = 282) | Experimental study with a 2 (low vs. high amount of information) X 3 (various levels of interactivity) between-subjects factorial design using an interactive TV ad. Outcome measures included brand attitude and recall (recorded by questionnaire) and time spent in the dedicated ad location (automatically recorded).  *Exposure:* Telescopic ad (30-second TV ad with “click the red button for more information” call to action and an interactive dedicated ad location) for a Dutch travel agency. The amount of information in the dedicated ad location was manipulated as either high or low. The interactivity was manipulated as 3 levels (varied by number of clickable links [12 vs. 92], presence/absence of navigation bars, option of 2-way communication) but was nonhierarchical. Participants entered an experimental “living room setting” and were shown the different interactive controls. Participants watched a 6-minute TV program excerpt and then the telescopic ad was shown. Participants could spend as much time in the dedicated ad location as they wanted. | [RQ 3](#T3Caubergheetal2010) | |
| Cho [33] | To explore factors influencing the clicking of online banner ads on the World Wide Web. | Nonrandom sample (N = 756) recruited from 134 education- and internet-related listservs. from CataList (a catalog of listserv lists across 11 countries—countries not specified) | Experimental analysis of a 2 (websites: book review/movie review) X 3 (counterbalanced banner presentation order) exposure to fictitious websites with banner ads. Outcomes evaluated included actual click-through rates on ads and other outcomes not eligible for this review (level of involvement, attitude toward ads, attitude toward websites).  *Exposure:* Book review and movie review websites and 2 fictitious product online banner ads were created. Content of websites: still photo of the movie/book, a review of the movie/book, and other information (e.g., director, actors, publisher). Each website had 3 banner ads: 2 study banner ads and a computer product dummy ad. Presentation order of banner ads was counterbalanced to prevent order effects. On the first 3 web pages, there were 5 hyperlinks (banner ad, links to other pages on website, and “next” button). Participants could view a maximum of 15 web pages on each website if they clicked all hyperlinks and a minimum of 3. | None | |
| Cho, Lee, and Tharp [35] | To examine the possibility that the degree of forced exposure in web advertising may affect consumers’ advertising perceptions, clicking behavior, attitudes toward the ad and the brand, as well as purchase intentions. | Members (N = 215) recruited from 40 listservs. related to education, internet, advertising, and marketing that are part of CataList (a catalog of listserv lists across 11 countries—countries not specified) | Experimental study of randomized exposure to 4 banner ads with varying levels of forced exposure. Participants completed a self-reported online survey related to click-through rates and ad recall and attitude.  *Exposure:* Four animated banner ads for 4 imaginary new brands for financial services (loans), travel, a computer, and a retailer. The ads had varying levels of forced exposure characterized as forced exposure with no skip option, forced exposure with skip option, pop-up window, and normal banner; each banner ad included (1) animation with 8 frames, (2) stimulation of consumer needs by showing problems and solutions, and (3) use of “click now” or “click here” at the end of the frame. | None | |
| Chung and Zhao [36] | To explore theoretically and empirically the effects of consumers’ website surfing behaviors on advertising effectiveness. | Undergraduate and graduate students (N = 180) from a large southern US university | 2 (personal motivation: low/high) X 3 (number of hyperlinks: 6, 24, 48) between-subjects experimental design of exposure to a fictitious product (camera) website. Outcomes included automatically recorded clicking behavior and movement during web surfing using software (not specified) embedded in the website, memory of product information (name, features) based on open-ended written recall then coded into a memory score consisting of 1 point for every product-related fact written, and other outcomes not eligible for this review (perceived interactivity, attitude).  *Exposure:* Fictitious website for a 35-mm point-and-shoot camera and digital camera. The number of hyperlinks on the website was manipulated into 3 levels (6, 24, 48) to offer users different ability to access information. Level of pages in the website was controlled to keep the amount of information available constant; font, page length, and other visual components were consistent across stimuli. | [RQ 3](#T3Chungetal2004) | |
| Graham et al. [30] | To demonstrate how online advertising can increase consumer demand for smoking cessation treatments, to show what tools can track and evaluate the impact of this kind of advertising, and to highlight challenges and future directions for research. | Individuals in Minnesota and New Jersey (N = NR) aged 18 and older with an interest in smoking cessation treatment content on the web | Observational analysis comparing online advertising (banner ads and paid search ads with embedded call to action) vs. traditional approaches (billboards, TV and radio ads, outdoor signage, direct mail, physician detailing) over 2 periods (5 months and 2 months). Measures of advertising performance included response rates, treatment enrollment, online ad tracking data, phone and web registration data, and QuickNet utilization data (number of log-ins and page views, session length, long-term cookies). Results were analyzed using descriptive statistics.  *Exposure:* Four online ads total; 3 were banner ads with different creative messages placed on national and local websites (e.g., Yahoo!, AOL, Weather.com), and the fourth was a paid search (text) ad; ads included a call to action (“click here”). When clicked, it took the user to the Healthways QuickNet landing page, where users could choose from 3 cessation treatment options. There was no interactivity or manipulation of information flow within the ad itself. Traditional ads were run in Minnesota and New Jersey with nearly identical content to the online ads as billboards, TV and radio ads, outdoor advertising (e.g., bus sides and shelters), direct mail, and physician detailing. Traditional ads contained the QuickNet URL. | [RQ 2](#T2Grahametal2008)  [RQ 4](#T4Grahametal2008) | |
| Horrell et al. [24] | To assess individuals’ engagement with Facebook ads designed to increase the number of persons opting into an online community related to lung cancer. | Users (N = 91 385) of Facebook between August and December 2017 aged 18 years and older with an interest in lung cancer–related content or Facebook pages | Observational analysis of 5 weeklong Facebook campaigns; each campaign included 3 unique ads. Measures of advertising performance included impressions (number of times the ad appeared in news feeds), reach (the number of individuals exposed to the ad), and user engagement, defined as interacting with the ad through clicks (reacting to the post, clicking a post link, or liking the Facebook page; sharing or commenting on the ad; or opting in and signing up for the online health community). Data source for measures was presumably from Facebook platform for advertisers but not explicitly stated.  *Exposure:* Each of 5 weeklong Facebook campaigns included 3 unique ads containing an image, text, and a call to action; the visuals included 6 static images and 1 image in graphic interchange format. | [RQ 2](#T2Horrelletal2019) | |
| Kim et al. [28] | To examine whether exposure to Tips From Former Smokers 2012 campaign digital ads influenced information-seeking behaviors online. | Data from comScore’s web panel of 1 million US adult internet users; the study included 15,319 participants exposed to a Tips 2012 campaign ad and 15,319 participants not exposed to the ad who were matched on demographics and internet use behavior | Observational study of participants recruited from a market research panel of web users to compare click-through rates of those exposed to digital ads for a smoking cessation website compared with a control group that was not exposed to the ads. Proportions of panelists who visited the Tips 2012 campaign website, the Smokefree.gov cessation site, and other noncampaign-related cessation sites or who searched for campaign-related topics and general information about cessation. Outcomes were calculated separately at each weekly time period after initial ad exposure.  *Exposure:* The Tips 2012 digital campaign included display, video, mobile, and search ads that were intended to direct smokers to the Tips 2012 website. Display ads were animated or static and appeared at the top or sidebar of popular websites to attract target audiences. Display ads were placed on select websites and ad networks and highlighted the stories of former smokers. Video ads ran on 29 websites and featured the same 30-second ads that ran on TV. Search ads appeared at the top and sidebar of search results so that when consumers typed in any of the paid search terms (e.g., how to quit smoking) on Google and Yahoo, the top result retrieved is the Tips 2012 site. All digital ads ran from March 19 to June 10, 2012. | [RQ 2](#T2Kimetal2016) | |
| Levy and Nebenzahl [38, 55] | To examine how interest in TV program content and category of product affects viewers' interactive behavior with commercial break interactive TV ads. | Israeli university students (N = 243) with past experience with the internet or other interactive media | Experimental design with interactive television commercials (2 television program segments and 4 paired sequences of commercials) among adult students. Two measures of interactive behavior, the number of times a respondent clicked on an interactive menu item, and the total time spent in interactive behavior with each commercial were automatically recorded by the interactive platform. Participants completed an online survey after exposure assessing level of product involvement, interest in getting additional product information, and purchase process information.  *Exposure:* Each student watched a short edited video recording of a television program with embedded commercials. Eight 12-minute short films comprising a program and 2 commercials were produced. Each started with a 5-minute program segment, followed by a commercial break, continuing with a second 5-minute program segment and ending with the second commercial break. Test ads were created for the following type of products/services: search products (jean pants), durable products (passenger car], nondurable products (perfume), credence products (car repair service). Respondents were randomly assigned to 1 of the 8 video versions in a 2 (TV program segment) X 4 (sequence of commercial pairs: search-durable, durable-credence, credence-nondurable, nondurable-search) design. The programs were viewed on a computer with the mouse replacing the remote control. The interactive platform included HTML pages, where respondents could get additional information, make a purchase decision, or exit the interactive platform. | None |  |
| Moe [39] | To explore the effects of timing on users’ direct and indirect response to promotional pop-up ads. | Users (N = 128,090) of an existing informational internet site | Five-day experimental study that manipulated pop-up ad timing on a real-world informational website across 3 dimensions over 4 days: across-page delay, within-page delay, and time of day. Clickstream data collected via cookies on the visitor’s computer recorded every page viewed and date and time of view, and each page view was associated with a unique session number defining a session consisting of consecutive pages viewed no more than 1 hour apart. On the fifth day, no promotions were offered, and users from this day represent the control group. Outcomes included click-through rates, page views, and variation by manipulated dimensions (page delays and time of day) and context of page (gateway or content page) where ad appeared.  *Exposure:* Real-world users of a movie information website that provides information about movies in theaters and on DVD, including critic reviews, trailers, and actor biographies were presented with a promotional pop-up ad offering subscription to a weekly newsletter. Timing of the ad was manipulated in 3 ways: across-page delay (presented on the first, second, or fourth page of their session), within-page delay (presented after 0, 15, or 30 seconds), and time of day (daytime, evening, or nighttime). The exposure was implemented over 4 consecutive nonholiday weekdays, and 1 additional day on which exposure was not implemented served as a control. Of 83,136 visitors to the website who were eligible to receive the ad, 38,834 received the ad (those who were eligible but did not receive the ad were assigned to an experimental condition that did not trigger the ad (e.g., visitor left before the fourth page or before the 30-second delay). | [RQ 2](#T2Moeetal2006) | |
| Munoz-Leiva, Hernández-Méndez, and Gómez-Carmona [26] | To assess advertising effectiveness of 3 Travel 2.0 tools/websites by measuring visual attention (via eye tracking) and recall (via post-exposure survey). | Social network users (N = 60) over age 18 years from the general public in Spain recruited via email and phone | Experimental within- and between-group subjects study during which participants viewed 3 websites with an embedded banner ad. Subjects viewed the websites in a random order and performed a task (navigate to find hotel room information). Their eye-tracking fixation counts, fixation durations, and time of first fixation were measured for each website area of interest. Participants reported level of ad recognition/recall in a post-exposure questionnaire.  *Exposure:* Replicas of 3 hotel “Travel 2.0 websites” (1 blog, 1 Facebook page, 1 Tripadvisor page) with an embedded vertical airline banner ad (“We fly just for you! Visit” with URL and incentive “for the chance to win prizes every week” with an image of celebrities with plane). Website was divided into multiple areas of interest (e.g., header, posts, comments section, banner ad, bottom of page) for the eye-tracking analysis. | [RQ 3](#T3MunozLeivaetal2019) | |
| Noel and Babor [27] | To determine if user engagement values and user-generated comments displayed alongside alcohol ads on social networking sites can increase ad engagement and the desire to drink. | Young adults (N = 120) recruited through Amazon Mechanical Turk. Key selection criteria: living in US, internet access, age 21 to 24. Two-stage recruitment procedure: screening survey and full survey. | 2 (regulatory compliant vs. noncompliant) X 2 (low vs. high user engagement) vs. 2 (pro- vs. anti-alcohol user-generated comments) factorial experiment among young adults randomized to view 4 alcohol ads within 1 of the 4 test conditions sequentially. Measures: desire to drink (assessed by 1 Likert question) and self-reported ad engagement defined on level of agreement with whether they would like or share the ad post.  *Exposure:* Used 2 Facebook ads for Bud Light and 2 Facebook ads for Budweiser. For each brand, 1 ad was compliant with the regulations and the other was noncompliant. Noncompliant ads promoted excessive alcohol consumption or associated alcohol use with success; compliant ads focused on product quality or promoted a TV commercial. Ads were then varied on 2 other factors: low vs. high user engagement defined as the number of likes, shares, or comments associated with the ad and pro- vs. anti-alcohol user-generated comments associated with the ad. | None | |
| Rauwers, Voorveld, and Neijens [42] | To investigate whether and how consumers use the interactive features placed in digital magazines ads and to explain the persuasive impact of interactive magazine ads on consumers’ ad and brand attitudes. | Dutch participants recruited through social media and email sent by a magazine publisher to 40,000 magazine consumers; recruits were required to use an Android device and were offered a free 8-week subscription to a a digital app for the magazine Flair (a Dutch women's magazine); 247 respondents met criteria, but 149 did not complete the study because of dropout (N=36) or because of not opening the magazine pages (N=113), leaving 98 participants in analyzed sample | Experimental study of volunteers receiving weekly digital magazine issues for 8 weeks who were randomized after the third issue to exposure to issues containing an ad with an interactive movie clip or the same ad without the interactive clip. Results were obtained through (1) an in-app analytics tool that created a dataset and analyzed with Python scripts to provide movie clip feature use, ad exposure time, movie clip exposure time, and app opening and (2) a post-exposure survey assessing general attitudes about the digital magazine and recall of the specific ad, attitude about the ad and the movie clip, and perceived interactivity.  *Exposure:* Participants were exposed to a magazine app that mimicked a hard copy magazine. Ad interactivity was manipulated by either integrating or omitting an interactive movie clip feature within a 2-page digital magazine ad for the brand Chanel. The movie clip feature was designed to be similar to a YouTube clip (e.g., containing a play button) and displayed a screenshot of Chanel’s TV commercial. When the playbutton was clicked, the commercial played full screen for a maximum duration of 196 seconds. | None | |
| Soneji et al. [25] | To assess changes in engagement with online tobacco and e-cigarette marketing among adolescents in the US between 2013 and 2015. | US youth age 12 to 17 sampled in Wave 1 (2013-2014; n = 13,651) and Wave 2 (2014-2015; n = 12,172) of the nationally representative PATH study | Observational study that investigated real-world engagement with 6 forms of online tobacco marketing between 2013 and 2015 in the nationally representative PATH study; data were collected through audio, computer-assisted self-interviews available in English and Spanish. Results were estimated using the weighted prevalence of affirmative responses of each form of engagement with online tobacco marketing and stratified by tobacco use category.  *Exposure:* Investigated engagement with 6 forms of online tobacco marketing: (1) signing up for email alerts, reading articles online, or watching videos online about tobacco products; (2) liking or following a tobacco brand on social media; (3) sending a tobacco brand link or information on social media sites; (4) playing online games related to tobacco brands; (5) receiving discount coupons electronically; and (6) receiving tobacco-related information electronically. | None | |
| Trappey and Woodside [37] | To examine consumer response to SMS-TV marketing (SMS messages advertising particular TV programs) (outbound) and consumer response to TV commercials that tell consumers to send SMS messages to advertising entity (inbound). | US and UK consumers from DataMiners Ltd. database (N varies by analysis: N = 5,401 UK households, N = 800 US consumers in a post-campaign evaluation, N = 2,200 UK consumers) who received SMS-TV marketing | Observational study involving analysis of real-world data recorded by DataMiners Ltd. Results from:  (1) 26 research consumer surveys evaluating UK SMS ad campaigns.  (2) Research consumer surveys evaluating 4 outbound campaigns from the US where consumers were directed to a particular TV channel or program.  (3) Dataminers.co.uk Mobile Media database, which includes phone interviews with UK consumers. Interviews asked about mobile phone usage, activity, attitudes, lifestyles, and behaviors.  Measures: Self-report of actions taken by consumers in response to SMS campaigns (e.g., watched TV, replied to SMS, bought advertised product, visited advertised website).  *Exposure:* SMS messages sent to consumers advertising a TV channel/program and TV ad campaigns with call to action for viewers to send SMS message to advertiser. TV channels/programs, products, and ads not specified. Ads not designed or implemented by study authors. | None | |
| Urban et al. [47] | To examine the performance of banner morphing on CNET’s website, explore interactions with context matching, and test whether banner morphing increases brand consideration and purchase likelihood. | Active consumers on CNET’s website from 4/11/2011 to 5/13/2011 (N = 116,168) | Experimental study with website users randomly assigned to test (70%) and control (30%) groups. Morphing algorithm automatically and optimally assigned banners to consumer segments (some context-matched, some were not). Measure: Click-through rate  *Exposure:* Eight banners advertised AT&T smartphones; some were context-matched and some were not. Five banners were square and could appear anywhere on the page; 3 banners were rectangular and appeared at the top of the page. Characteristics of the morphs varied: smartphone image, size of image, size and colors of font, background colors, information content, and hot links. Morphing involves a dynamic change in the ad to match the latent cognitive style that is inferred from a consumer’s clickstream behavior. | [RQ 2](#T2Urbanetal2014) | |
| Yang [53] | To compare the effectiveness of interactive and noninteractive ads. | Taiwanese, Chinese, and American graduate students at a university in the Midwest US (N = 108, with 36 participants from each nationality) | Experimental study where participants were exposed to interactive and noninteractive ads for batteries and floppy disks in a simulated online store. All participants viewed all 4 ad categories (battery-interactive, battery-noninteractive, floppy disk-interactive, floppy disk-noninteractive) but viewed them in 1 of 4 orders. After each ad, participants completed survey questions (pen and paper and online). Outcome measures included time spent on each ad measured by the accumulative seconds each subject spent watching each test ad tracked by the computer and attitude toward the ad and brand measured through post-exposure surveys.  *Exposure:* Seven pairs of ads, all with similar layouts and consistent with late 1990s major online store message design strategies. Each pair contained 2 ads with the same amount of product information (same number of frames/pages): 1 noninteractive ad (no control over presentation of information) and 1 interactive ad (participants chose how long to watch, how long to view each page, branch of information to view, depth of information hierarchy to view, order of information, ability to skip ahead). Ads were for batteries or floppy disks. | [RQ 3](#T3Yangetal1997) | |
| Yi, Jiang, and Benbasat [46] | To investigate the effects of 3 online product presentation formats (noninteractive video, full interaction, restricted interaction) on engaging online users and enticing them to pursue the product offline. | Students and staff (N = 170) recruited from a major public university (country not specified) | Experimental study using a fictitious cell phone brand/model as the targeted product modeled after the Nokia Symbian. Participants randomly assigned to 1 of 3 presentation conditions and asked to examine the product website as if they were shopping online. Outcomes measured through a post-experimental questionnaire focusing on measures of enticement and engagement (i.e., attention and focus on the product presentation during the experiment) of online product experience and intentions to purchase.  *Exposure:* Website for a fictitious cell phone with 4 functions (calls/contacts management, keyboard settings, photo display editing, gaming) modeled after the Nokia Symbian and presented in 1 of 3 presentation formats. Factual product information was uniform across conditions. Noninteractive condition (N = 55): viewed prerecorded videos about the product functions. Full interaction condition (N = 58): launched virtual product experience simulators from the product homepage to sample functions of the cell phone using the mouse. Restricted interaction condition (N = 57): interacted with virtual product in limited steps; active participation followed by video demonstrations. | None | |
| Yoo [40] | To understand the effects of incidental exposure to web ads on memory, attitude toward the ad, brand consideration, and click-through intention and to investigate the conditions most conducive to incidental web ad exposure. | Experiment 1: undergraduate US students from an introductory advertising class (N = 180)  Experiment 2: undergraduate US students (N = 124) | Experiment 1 tested effects of incidental exposure to web ads on priming with participants randomly assigned to 1 of 3 groups: (1) incidental exposure, (2) full attention, or (3) no exposure. Participants viewed all 3 web pages for 2 minutes each and were asked to summarize web page content. Measures: Implicit memory, click-through intention  Experiment 2 assessed the conditions conducive to incidental exposure by examining 2 advertising strategies—format and placement of ads—with participants randomly assigned to either control or 1 of 4 experimental conditions (animated vs. static X congruency vs. incongruency). Measures: Priming. Ad recall and ad recognition measured for manipulation checks. Other outcomes not eligible for this review were also measured in both experiments.  *Exposure:* Experiment 1: 3 banner ads (1 target and 2 filler) were developed for fictitious brand names and placed at the top of 3 web pages that were developed (web pages were for a computer buying guide, a movie review, and a travel guide).  Experiment 2: The same 3 web pages were used as in Experiment 1. The same static banner was used from Experiment 1 plus an animated target banner ad. | None | |
| Yoo [41] | To examine the effects of message framing, keyword insertion, and users’ level of product involvement on keyword search ad click-through behaviors. | Undergraduate US students (N = 166) recruited from a communications class | Experimental study using 2 (message framing: negative vs. positive) X 2 (keyword insertion: yes vs. no) design with ads for a fictitious textbook brand displayed in response to “discount” search term typed in fabricated search engine. Users completed a product involvement survey beforehand and were assigned to 1 of 4 experimental conditions, entered “discount” into the search engine, then interacted with the page. Measures were click-through behavior (whether ad was clicked or not) and a post-experiment survey of perceived ad relevance.  *Exposure:* Four target ads for a fictitious textbook website (books chosen for medium product involvement): 2 (message framing: negative vs. positive) X 2 (keyword insertion: yes vs. no) presented after users instructed to type a specific search term (“discount”) into a stimulus search engine. Subjects were then shown 1 of 4 versions of the search engine results page that included 3 keyword search ads in a highlighted box labeled as sponsored links and 10 organic (unpaid) links. The target ad was located in the second position of the 3 keyword search ads. Subjects were then asked to indicate their normal click-through behaviors by clicking a checkbox located next to a hyperlink. The ads displayed were text only. | None | |

Abbreviations: CNET, computer network (an American media site); DVD, digital versatile disk; HTML, hypertext markup language; N, number; NR, not reported; PATH, Population Assessment for Tobacco and Health study; RQ, research question; SMS, short message service; SMS-TV, short message service TV; TV, television; UK, United Kingdom; URL, Uniform Resource Locator; US, United States; vs., versus.

**Table S4-3. Extent of Engagement in Interactive Ads by Consumers in Naturalistic Contexts (Research Question 2)**

| Author (year) | Study description | Exposure | Findings | Other relevant RQs |
| --- | --- | --- | --- | --- |
| Birnbaum et al. [29] | Observational study evaluating the effect of Google AdWords ads directed toward help-seeking individuals with early-stage psychotic disorders in New York City metro area (N = NR). | 154 unique ads were created, cued by more than 2,000 relevant search terms (containing words like “psychosis,” “hearing voices,” “mind control,” “bipolar disorder”) and organized across 15 thematic ad groups; each ad contained a headline (thematically related to the user’s search term), a description line (supporting information), and a call to action (encouraging users to click the ad, take a quiz, find support, etc.). Unclear whether users were able to get more information or take a quiz within the ad itself or if they were immediately directed to the landing page for the early treatment program. | Number of times ad appeared: 191,313  Number of times ad was clicked: 4,350 (2.3%)  Median session length on landing page of early treatment program website: 51 seconds (IQR 74 to 115)  Median page views at early treatment program website: 1.29  Number of users who clicked further into the site of those who clicked the ad: 1,918 (44%)  Number of users who completed psychosis screener of those who clicked the ad: 671 (15%)  Number of users who contacted the early treatment program directly of those who clicked the ad: 57 (1%) | [RQ 1](#T1Birnbaumetal2017) |
| Chonguy and Yuxing [54] | Observational study of daily Google AdWords data from 13 different advertisers using paid search advertising campaigns to investigate differences in consumer (N=NR) click-through behavior and differences between platforms (i.e., desktop, smartphone, tablet). | Paid search ads using Google AdWords from 13 advertisers from 1/1/2015 to 12/31/2016; advertisers belonged to industries including health care, technology services, construction, and manufacturing among others. | Range across advertisers  Number of ad impressions: 87,702 to 8,308,158  Number of ad clicks: 1,817 to 291,964  Average click-through rate: 0.02 to 0.15 | None |
| Graham et al. [30] | Observational study comparing impact of online advertising with traditional approaches for recruiting individuals (N = NR) to smoking cessation treatment. Ads used creative messaging and directed viewers to the QuickNet website to register for cessation treatments. | Four online ads total; 3 were banner ads with different creative messages placed on national and local websites (e.g., Yahoo!, AOL, Weather.com), and the fourth was a paid search (text) ad; ads included a call to action (“click here”). When clicked, it took the user to the Healthways QuickNet landing page, where they could choose from 3 cessation treatment options. There was no interactivity or manipulation of information flow within the ad itself. Traditional ads were run in Minnesota and New Jersey with nearly identical content to the online ads as billboards, TV and radio ads, outdoor advertising (e.g., bus sides and shelters), direct mail, and physician detailing. Traditional ads contained the QuickNet URL. | Number of individuals seeking information at website during campaign: 130,214  Number of online ad clicks: 106,291 (81.6% of those seeking information at website during campaign)  Number of individuals who registered for any form of cessation treatment after click-through online ad: 9,655 (9.1% of those who clicked through online ad)  Number of individuals who registered for treatment on website as a result of traditional media: 23,923 (18.6% of those seeking information at website during campaign) | [RQ 1](#T1Grahametal2008)  [RQ 4](#T4Grahametal2008) |
| Horrell et al. [24] | Observational study of user (N = 91,385) engagement with 5 weeklong Facebook advertising campaigns each including 3 unique ads all seeking to increase membership in an online community related to lung cancer. | Each of the 5 weeklong Facebook campaigns included 3 unique ads containing an image, text, and a call to action; the visuals included 6 static images and 1 image in graphic interchange format. | Number of individuals exposed to ad: 91,835  Number of reactions: 2,602 (2.80%)  Number of link clicks: 1,789 (1.90%)  Number of page likes: 149 (0.16%)  Number of shares: 452 (0.50%)  Number of comments: 157 (0.17%)  Number of individuals opting into the online health community: 863 (0.94%) | [RQ 1](#T1Horrelletal2019) |
| Kim et al. [28] | Observational study of web users (N = 30,638) who were exposed or not exposed to ads for the Tips From Former Smokers 2012 campaign to assess whether exposure was associated with online searches or visits to campaign-related websites. | The Tips 2012 digital campaign included display, video, mobile, and search ads that were intended to direct smokers to the Tips 2012 website. Display ads were animated or static and appeared at the top or sidebar of popular websites to attract target audiences. Display ads were placed on select websites and ad networks and highlighted the stories of former smokers. Video ads ran on 29 websites and featured the same 30-second ads that ran on TV. Search ads appeared at the top and sidebar of search results so that when consumers typed in any of the paid search terms (e.g., how to quit smoking) on Google and Yahoo, the top result retrieved was the Tips 2012 site. All digital ads ran from March 19 to June 10, 2012. | Percentage of participants with visits to Tips 2012 site; Wk1, Wk2, Wk3, Wk4  Exposed: 0.4%, 0.6%, 0.8%, 0.9%  Unexposed: 0.1%, 0.2%, 0.3%, 0.4%  *P* < .001 for each week  Percentage of participants with visits to any non-Tips 2012 cessation sites; Wk1, Wk2, Wk3, Wk4  Exposed: 0.2%, 0.2%, 0.3%, 0.3%  Unexposed: 0.0%, 0.1%, 0.1%, 0.2%  *P* = .001 for Wk1 and Wk2, *P* = .005 for Wk3, *P* = .019 for Wk4  Percentage of participants who conducted search on campaign-related terms; Wk1, Wk2, Wk3, Wk4  Exposed: 0.2%, 0.4%, 0.6%, 0.7%  Unexposed: 0.2%, 0.3%, 0.4%, 0.5%  *P* = .414 for Wk1, *P* = .066 for Wk2, *P* = .032 for Wk3, *P* = .122 for Wk4  No significant difference in percentage of participants who visited the Smokefree.gov website (<0.1%) between exposed and unexposed (actual values and data NR). | [RQ 1](#T1Kimetal2016) |
| Moe [39] | Experimental study of real-world users (N = 128,090) of an informational website about movies that assessed the impact of timing of pop-up ads offering subscription to a weekly email using click-through rates and page views. | Real-world users of a movie information website that provides information about movies in theaters and on DVD, including critic reviews, trailers, and actor biographies, were presented with a promotional pop-up ad offering subscription to a weekly newsletter. Timing of the ad was manipulated in 3 ways: across-page delay (presented on the first, second, or fourth page of their session) and within-page delay (presented after 0, 15, or 30 seconds) and time of day (daytime, evening, or nighttime). The exposure was implemented over 4 consecutive nonholiday weekdays and 1 additional day where exposure was not implemented served as a control. Of 83,136 visitors to the website who were eligible to receive the ad, 38,834 received the ad (those who were eligible but did not receive the ad were assigned to an experimental condition that did not trigger the ad [e.g., visitor left before the fourth page or before the 30-second delay]). | Overall click-through rate for pop-up ad: 0.81% (314 of 38,834 visitors who received a pop-up ad)  Click-through rate (0-second ad delay): 1.14%  Click-through rate (15- and 30-second ad delay): 0.65%  *P* > .0001 for 0-second vs. 15- or 30-second delay  *P =* NS comparing click-through rates for across-page delay (actual rates NR) and context of page (gateway page vs. content page)  Mean number of pages viewed (ad appeared on a gateway page): 4.86  Mean number of pages viewed (ad appeared on content page): 6.31  *P* < .001  Mean number of pages viewed (0-second delay): 6.39  Mean number of pages viewed (15- or 30-second delay): 5.61  *P* = .0295  Mean number of pages viewed (experimental group): 5.86  Mean number of pages viewed (control group not exposed to ad): 3.45  *P* < .001  The following results used a conditional control sample to control for the fact that the across-page delay condition only affected those site visitors who view at least as many pages as those in the delay condition*  Mean number of pages viewed (ad on first page): 3.06  Mean number of pages viewed (conditional control sample): 3.45  *P* < .0001  Mean number of pages viewed (ad on second page): 7.53  Mean number of pages viewed (conditional control sample): 7.07  *P* = .0001  Mean number of pages viewed (ad on fourth page): 11.66  Mean number of pages viewed (conditional control sample): 11.59  *P* = .3578 | [RQ 1](#T1Moe2006) |
| Platt et al. [31] | Observational analysis of Facebook ad campaign targeted to Michigan residents age 18 to 28 (N = ~2 million) to promote awareness of state biobank and evaluated with engagement measures. | Twenty-six–day Facebook ad campaign with the goal of appealing to the user’s connection to Michigan either in the text or by incorporating the shape of the state into the associated image. Twenty-seven ads were used in total: 17 promoted a photo contest or its prizes, 5 provided an educational message or the promise of a message (e.g., “Click here to learn about…”), 3 appealed to an individual’s affiliation with a school that was visited as part of the campaign, and 2 “sponsored” ads used social connections to draw attention (e.g., “your friend likes this”). Viewers who clicked on an ad were redirected to 1 of 3 landing pages (biobank campaign website, biobank Facebook page, page for photo contest). Ads were created and revised to respond to performance in real time. The campaign ran concurrently with 12 college campus visits and with the submission phase of the “mybloodspot.org” photo contest that also ran on the biobank’s Facebook page. | Number of ad impressions: 20,087,795  Number of users viewing ads: 779,004 (each user viewed an ad an average of 25.8 times)  Number of biobank Facebook page views: 1249 during the month of the campaign vs. once in the month prior vs. 813 in the month after  Number of users clicking ads: 4,275 (click-through rate 0.021% based on impressions)  Number of users who clicked through to the most detailed information on the website: 200 (4.7% of those who clicked on an ad)  Number of biobank Facebook page or post likes: 572 during month of the campaign (516 of which were after viewing an ad) vs. once in the month prior vs. 69 in the month after  Number of users adding a post or commenting on a post on the biobank Facebook page: 3  Number of users participating in photo contest: 30 Researchers found that viewers were far more likely to click an ad or post they saw associated with the name of a friend who had already liked the Facebook page. A quarter of visitors to the homepage went on to look at a second page of the website, and 14% looked at a third page; a third of visitors spent more than a minute on the website, and 12.4% spent more than 10 minutes. | None |
| Urban et al. [47] | Experimental study to examine the performance of banner ad morphing and explore context matching with active consumers on CNET website (N = 116,168) who were randomized to test and control groups over the period of a month. | Eight banners advertised AT&T smartphones; some were context-matched naturally (i.e., not manipulated) and some were not. Context-matching refers to the ads appearing on CNET pages where any smartphone was rated, compared, or priced. Five banners were square and could appear anywhere on the page; 3 banners were rectangular and appeared at the top of the page. Characteristics of the morphs varied: smartphone image, size of image, size and colors of font, background colors, information content, and hot links. Morphing involves a dynamic change in the ad to match the latent cognitive style that is inferred from a consumer’s clickstream behavior. | Number of times banner ads appeared: 451,524  Number of times banner ads appeared that were context-matched: 58,899 (13%)  Number of individuals exposed to banner ads: 116,168  Number of individuals exposed to context-matched banner ads: 32,084 (27.4%)  Click-through rate (all banners)  Context matched  Morphing: 0.31%  Control: 0.17%  83% relative increase; *P* = .003  Not context matched  Morphing: 0.15%  Control: 0.16%  6% relative decrease, *P* = .495  Morphing X context-matching interaction; *P* < .01  Click-through rate (per consumer)  Context matched  Morphing: 0.25%  Control: 0.13%  97% relative increase, *P* = .028  Not context matched  Morphing: 0.14%  Control: 0.20%  27% relative decrease, *P* = .081  Morphing X context-matching interaction; *P* = .017 | [RQ 1](#T1Urbanetal2014) |

Abbreviations: CNET, computer network (an American media site); DVD, digital versatile disk; IQR, interquartile ratio; N, number; NR, not reported; NS, not significant; RQ, research question; TV, television; URL, Uniform Resource Locator; Wk, week.

**Table S4-4. Association Between Interactive Features and Selected Outcomes (Research Question 3)**

| Author (year) | Study description | Exposure | Findings | Other relevant RQs |
| --- | --- | --- | --- | --- |
| Cauberghe and De Pelsmacker [51] | Experimental study among participants (N=282) from a Belgian market research firm exploring the effects of differing levels of ad interactivity and information within an interactive TV ad on user brand attitudes, recall, and time spent in the dedicated ad location. | Telescopic ad (30-second TV ad with “click the red button for more information” call to action and a interactive dedicated ad location) for a Dutch travel agency. The amount of information in the dedicated ad location was manipulated as either high or low. The interactivity was manipulated as 3 levels (varied by number of clickable links [12 vs. 92], presence/absence of navigation bars, option of 2-way communication) but was nonhierarchical. Participants entered an experimental “living room setting” and were shown the different interactive controls. Participants watched a 6-minute TV program excerpt and then the telescopic ad was shown. Participants could spend as much time in the dedicated ad location as they wanted. | Time (minutes) spent in dedicated ad location  Low interactivity: 4.4  High interactivity: 6.1  *P* < .001  Interaction with amount of information  Low information  Low interactivity: 3.4  High interactivity: 6.1  *P* < .001  High information  Low interactivity: 5.0  High interactivity: 6.3  *P* = .152  Brand recall  Low interactivity: 43.6%  High interactivity: 56.4%  *P* = .02  Interaction with amount of information:  Low information  Low interactivity: 41.1%  High interactivity: 58.9%  *P* = .029  High information  Low interactivity: 46.3%  Low interactivity: 53.7%  *P* = .273  Relationship between factors and brand recall (multivariate logistic regression)  Time spent in dedicated ad location; *P* = .041, more time was associated with higher recall  Attitude toward brand; *P* = .015, more favorable attitude was associated with higher recall  Level of interactivity; *P* = .122, no relationship  Amount of information; *P* = .383, no relationship  Interaction between interactivity and amount of information; *P* = .673 | [RQ 1](#T1Caubergheetal2010) |
| Chung and Ahn [32] | Undergraduate US students (N = 181) participated in a 2 (personality difference) X 4 (website structure) experimental study examining the most effective web ad structure on memory, attitude, and behavioral intentions. | A website for purchasing computers with 10 pages. Linear structure: Scroll to bottom of page and click a button to move to the next page. Interactive structure: Click any link on page. Mixed structure: 5 pages linear, 5 pages interactive. Participants not allowed to click the back button. | Mean product memory score  Linear: 2.66 (range of scale NR)  Interactive: NR  Mixed: NR  *P* < .01 across groups; linearly structured website had highest score and was significantly different compared with interactive website or mixed website | None |
| Chung and Zhao [36] | Experimental analysis of a 2 (personal motivation) X 3 (number of hyperlinks) exposure to fictitious product (camera) website with undergraduate US university students (N = 180) to explore the effects on website surfing behaviors and advertising effectiveness. | Fictitious website for a 35-mm point-and-shoot camera and digital camera. The number of hyperlinks on the website was manipulated into 3 levels (6, 24, 48) to offer users different ability to access information. Level of pages in the website was controlled to keep the amount of information available constant; font, page length, and other visual components were consistent across stimuli. | Mean product information memory score (SD): 1.46 (1.73)  Significant association between number of clicks (more) and memory score (higher); *P* < .001 | [RQ 1](#T1Chungetal2004) |
| Daems, De Pelsmacker, and Moons [43] | A 2 (integration vs. no integration) X 2 (brand interactivity vs. no brand interactivity) between-subjects experiment among Belgian secondary school youth (N = 576) examining the effect of online advertising’s brand interactivity and its integration in other content on young teenagers’ brand memory, awareness of selling intent, critical processing, brand attitude, and their personal information sharing. | All 4 target ads were for a fictitious smartphone (“Delta”). Two ads had integrated ads (i.e., advergame) involving a puzzle game. One of the 2 integrated ads involved brand interaction (i.e., manipulating game elements related to the smartphone, color, price, or capacity) as part of the advergame, and the other included a static noninteractive ad for the brand on the side of the screen (i.e., in-game advertising) for an advergame featuring brand integration for a filler ad (roller-coaster). Participants were given 70 seconds of game play. The 2 other Delta ads (nonintegrated) were banner ads on a website. The brand interactive condition consisted of 3 banners featuring Delta that users could click for further information shown in pop-ups, which included different pictures with short messages providing more information about the brand (color, price, capacity) identical to the information in the advergame and a nonclickable ad for the roller-coaster. The noninteractive banner ad condition included 1 nonclickable ad for Delta featuring color, price, and capacity and 3 clickable filler ads for the roller-coaster. | Percentage of participants with brand recognition  Interactive banner ad: 60.4  In-game advertising ad: 22.4  Noninteractive banner ad: 21.0  Advergame: 14.3  Interactions  Recognition higher for nonintegrated formats vs. integrated formats, *P* < .001  Recognition higher for brand interactive formats vs. noninteractive formats *P* < .001  Integration X interactivity interaction NR  Mean (SD) memory of product characteristics (0 lowest, 12 highest)  Interactive banner ad: 8.22 (2.93)  In-game advertising ad: 4.12 (2.98)  Noninteractive banner ad: 3.87 (2.92)  Advergame: 5.79 (2.82)  Interactions  Memory higher for interactivity vs. noninteractivity; *P* < .001  Memory lower for brand integration vs. nonintegration; *P* < .001  Integration X interactivity interaction; *P* < .001 | None |
| Macias [44] | 2 (interactivity: low/high) X 2 (product: camera/athletic shoes) between-subjects experiment among US undergraduate students (N = 153) to evaluate how the level of ad interactivity in product websites influences comprehension and attitudes. | Participants viewed a low- or high-interactivity website for 1 of 2 brands (Nikon point-and-shoot camera and New Balance tennis shoes). Websites were created from print ads with enough information to make a website that allowed for interactive elements; within-brand sites were nearly identical in content. Low interactivity: Not adding any interactivity to print ad. High interactivity: Animation with mouse rollover, hyperlinks, comment forms, chat features | Mean (SD) comprehension score (0 min, 6 max)  High interactivity: 4.1 (NR)  Low interactivity: 4.0 (NR)  *P* = .029  Product involvement X interactivity interaction; *P* = .034, higher product involvement with high interactivity was associated with higher comprehension compared with low interactivity; lower product involvement with low interactivity was associated with higher comprehension compared with high interactivity.  Web experience X interactivity interaction *P* = .009  Higher web experience with high interactivity was associated with higher comprehension compared with low interactivity  Lower web experience with low interactivity was associated with higher comprehension compared with high interactivity. | None |
| Munoz-Leiva, Hernández-Méndez, and Gómez-Carmona [26] | Experimental study of banner ad effectiveness on Travel 2.0 websites. Spanish social network users (N = 60) viewed 3 websites with an embedded ad. Study used eye-tracking and self-report survey as measures of ad effectiveness and recall. | Replicas of 3 hotel “Travel 2.0 websites” (1 blog, 1 Facebook page, 1 Tripadvisor page) with an embedded vertical airline banner ad (“We fly just for you! Visit” with URL and incentive “for the chance to win prizes every week” with an image of celebrities with plane). Website was divided into multiple areas of interest (e.g., header, posts, comments section, banner ad, bottom of page) for the eye-tracking analysis. | Mean (SD)  Number of fixations on ad  Blog: 11.7 (8.3)  Facebook: 19.1 (12.6)  Tripadvisor: 6.1 (10.3)  *P* < .001  Time to first fixation (seconds) on ad  Blog: 23.1 (18.4)  Facebook 14.1 (13.3)  Tripadvisor: 38.6 (28.3)  *P* < .001  Total fixation duration (seconds) on ad  Blog: 2.4 (1.9)  Facebook: 4.1 (3.1)  Tripadvisor: 1.2 (2.5)  *P* < .001 | [RQ 1](#T1MunozLeivaetal2019) |
| Polster et al. [34] | Experimental analysis of whether important safety information is noticed and recalled on mocked up websites for a fictitious asthma product among US patients with asthma from an online research panel (N = 1,600). | Four versions of mocked-up websites that differed in how ISI could be accessed and displayed for a fictitious product to treat asthma: 2 were noninteractive (versions A and B: ISI at the bottom of the landing page but presented in 2 different layouts) and 2 were interactive requiring participants to click on a link to see full ISI (version C was a call to “click for full ISI” and then information presented in a question-and-answer (Q&A) format and version D was a single statement of risk and call to click for more information presented in a traditional format). In addition, websites were presented for either a desktop or smartphone, resulting in 8 independent exposure groups. Platform presentation was not randomized but rather determined by the device used by respondents to reply to the survey invitation. | Percentage of participants seeing any ISI (as measured through objective clicking/scrolling behavior); percentage of desktop participants, percentage of smartphone participants  A-Noninteractive (ISI bottom of page layout 1): 98, 100  B-Noninteractive (ISI bottom of page layout 2): 83, 89  C-Interactive (click to see full ISI/Q&A): 44, 40  D-Interactive (risk + click for full ISI/traditional): 9, 22  *P* < .05 comparing C vs. D on smartphone platform, *P* < .001 for all other pairwise comparisons  Recall of at least 1 relevant side effect; percentage of desktop participants, percentage of smartphone participants  A-Noninteractive (ISI bottom of page layout 1): 78, 81  B-Noninteractive (ISI bottom of page layout 2): 73, 61  C- Interactive (click to see full ISI/Q&A): 52, 53  D-Interactive (risk + click to see full ISI/traditional): 35, 49  Significant difference across desktop participants *P* < .001  Pairwise comparisons among desktop participants: With the exception of A vs. B, all other pairwise comparisons were significant (*P* < .001 A vs. C, A vs. D, B vs. C, B vs. D; *P* < .01 C vs. D)  Significant differences among smartphone participants (*P* < .001; pairwise comparisons among smartphone participants: *P* < .001 A vs. B, A vs. C, A vs. D; all other comparisons were NS)  Recall of at least 1 relevant side effect among participants who saw any ISI based on objective clicking/scrolling behavior); percentage of desktop participants; percentage of smartphone participants  A-Noninteractive (ISI bottom of page): 79, 81  B-Noninteractive (ISI bottom of page layout 2): 77, 64  C-Interactive (click to see full ISI/Q&A): 85, 79  D-Interactive (risk + click to see full ISI/traditional): 83, 89  No significant difference across desktop participants  Significant differences across smartphone participants *P* < .001; pairwise comparisons among smartphone participants: (*P* < .01 A vs. B; *P* < .001 B vs. D; all other comparisons were NS)  Mean percentage correct recognition of product side effects (defined as correctly answering “yes” to all 12 side effects for the product); percentage of desktop participants, percentage of smartphone participants  A-Noninteractive (ISI bottom of page layout 1): 41, 46  B- Noninteractive (ISI bottom of page layout 2): 34, 37  C-Interactive (click to see full ISI/Q&A): 32, 35  D-Interactive (risk + click to see full ISI/traditional): 22, 30  Significant differences for desktop participants; *P* < .001; pairwise comparisons among desktop participants (*P* < .05 A vs. C; *P* < .001 A vs. D, B vs. D, C vs. D; all other comparisons were NS)  Significant differences for smartphone participants; *P* < .001; pairwise comparisons among smartphone participants (*P* < .01 A vs. B; *P* < .001 A vs. C, A vs. D; all other comparisons were NS)  Mean percentage correct recognition of product side effects (defined as correctly answering “yes” to all 12 side effects for the product) among participants who saw any ISI based on objective clicking/scrolling behavior); percentage of desktop participants, percentage of smartphone participants  A-Noninteractive (ISI bottom of page layout 1): 41, 46  B-Noninteractive (ISI bottom of page layout 2): 38, 38  C-Interactive (click to see full ISI /Q&A): 47, 46  D-Interactive (risk + click to see full ISI/traditional): 44, 36  No significant differences among desktop participants  Significant differences among smartphone participants *P* < .05; pairwise comparisons (*P* < .05 A vs. B; all other comparisons were NS) | None |
| Yang [53] | Experimental study among graduate students (N = 108) comparing the effectiveness of interactive and noninteractive ads by measuring average time spent in each ad. type and brand and ad attitude. | Seven pairs of ads, all with similar layouts and consistent with late 1990s major online store message design strategies. Each pair contained 2 ads with the same amount of product information (same number of frames/pages): 1 noninteractive ad (no control over presentation of information) and 1 interactive ad (participants chose how long to watch, how long to view each page, branch of information to view, depth of information hierarchy to view, order of information, ability to skip ahead). Ads were for batteries or floppy disks. | Mean (SD) time in seconds spent viewing ad  Interactive battery: 136.0 (118.7)  Noninteractive battery: 373.0 (451.3)  Interactive floppy disk: 135.3 (104.6)  Noninteractive floppy disk: 171.1 (62.3)  Interactivity main effect *P* < .01 (interactive ads were viewed for less time compared with noninteractive ads)  Product X interactivity interaction *P* < .01 | [RQ 1](#T1Yang1997) |

Abbreviations: ISI, important safety information; N, number; NR, not reported; NS, not significant; Q&A, question and answer; RQ, research question; SD, standard deviation; TV, television; URL, Uniform Resource Locator; US, United States; vs., versus.

**Table S4-5. Systematic Review Related to Association Between Interactive Features and Selected Outcomes (Research Question 3)**

| Author (year) | Study objective | Description of included studies | Findings |
| --- | --- | --- | --- |
| Yang and Shen [45] | To review the extent to which web interactivity can affect various psychological outcomes and how such effects are moderated. | 63 experimental studies (N = 13,484) with quantifiable data suitable for use in a meta-analysis on outcomes related to cognition, enjoyment, attitudes, or behavioral intention.   - 32 studies (51%) were focused on interactivity in an advertising context; the rest were focused on interactivity in a news, health, or political information context or in a learning/educational context. - 16 studies (25%) reported cognition outcomes (the only outcome of relevance for this scoping review). | Cognition was defined as “mental activities of information processing and storage” and included measures of comprehension, elaboration, knowledge acquisition, and recall.  No significant relationship was observed between interactivity and cognition (correlation coefficient = 0.050; *P* = .25). Additional analyses suggested no significant effect on comprehension, elaboration, or knowledge acquisition and a small negative effect on message recall. |

Abbreviations: N, number.

**Table S4-6. Comparison Between Noninteractive Ads and Interactive Ads on Selected Outcomes (Research Question 4)**

| Author (year) | Study description | Exposures | Findings | Other relevant RQs |
| --- | --- | --- | --- | --- |
| Bellman et al. [49] | Experimental study among Australian audience panel members (N = 233) comparing the effectiveness of PC advergames, TV commercials, and interactive commercials with advergames on brand attitude and day-after recall of ad design/content. | Randomization to 1 of 3 ad types for 4 test brands (foods and personal hygiene products); Group 1 = PC advergame developed specifically for each of the 4 test brands;  Group 2 = normal 30-second TV commercial during a half-hour sitcom for 4 test brands;  Group 3 = interactive TV ad consisting of the TV commercial for the test brand enhanced with the advergame played using the TV’s remote control. Two of the 4 advergames used a quiz format, and the other 2 were simple games requiring skill (1 was a variation of PacMan). Participants watched TV program or played advergames in a laboratory setting designed like viewing at home. | Mean (SD) percentage of participants interviewed whose response qualified as proven day-after recall defined as unaided recall of at least 3 unique points about the ad  Group 1 (PC advergame): 91.9 (19.4)  Group 2 (TV commercial): 54.3 (29.9)  Group 3 (interactive TV): 70.1 (29.7)  Group 1 vs. Group 2; *P* < .001  Group 1 vs. Group 3; *P* < .001  Group 2 vs. Group 3; *P* = NR | None |
| Graham et al. [30] | Observational study comparing impact of online advertising with traditional approaches for recruiting individuals in 2 US states (N = NR overall, N = 32,459 for this analysis) to smoking cessation treatment. Ads used creative messaging and directed viewers to the QuickNet website to register for cessation treatments. | Four online ads total; 3 were banner ads with different creative messages placed on national and local websites (e.g., Yahoo!, AOL, Weather.com), and the fourth was a paid search (text) ad; ads included a call to action (“click here”). When clicked, it took the user to the Healthways QuickNet landing page, where users could choose from 3 cessation treatment options. There was no interactivity or manipulation of information flow within the ad itself. Traditional ads were run in Minnesota and New Jersey with nearly identical content to the online ads as billboards, TV and radio ads, outdoor advertising (e.g., bus sides and shelters), direct mail, and physician detailing. Traditional ads contained the QuickNet URL. | Traditional recruitment engagement metrics  Median average session length:12 minutes (range 8 to 20)  Pages viewed: 40 (range 22 to 98)  Percentage posting 1 or more times in public forums: 11  Percentage who sent Qmail to 1 or more persons: 35.5  Percentage who received Qmail from 1 or more persons: 10  Online recruitment engagement metrics; P value vs. traditional approach  Median average session length: 12 minutes (range 8 to 19); *P* = .01 (lower than traditional ads)  Pages viewed: 35 (range 20 to 76); *P* < .001  Percentage posting 1 or more times in public forums: 8.3; *P* < .001  Percentage who sent Qmail to 1 or more persons: 28.1; *P* < .001  Percentage who received Qmail from 1+ persons: 6.4; *P* < .001  Compared with traditional ads, online ads recruited a higher percentage of males, young adults, racial/ethnic minorities, those with a high school education or less, and dependent smokers. | [RQ 1](#T1Grahametal2008)  [RQ 2](#T2Grahametal2008) |
| Ieva et al. [48] | Experimental study comparing digital flyer delivered via email vs. traditional print flyer among a random sample of retail customers from an Italian supermarket’s loyalty card database (N = 9,902 randomized and included in purchase behavior analyses, 1,222 of which participated in follow-up telephone survey assessing recall). | Supermarket ad flyer (valid for 14 days) had a length of 32 pages and promoted 268 items. The online flyer was a direct replication of the print flyer, with no banners, videos, or embedded links. The user could click on the pages to zoom or move to another page and could choose among different zoom levels (i.e., the same options as available for any common PDF file). A delivery agency delivered the print flyer to the participants’ specified addresses. The online flyer was delivered by an email message containing a link to the flyer. | Analysis conducted only among persons reporting that they received and viewed the flyer (N = 102 for online flyer; N = 101 for print flyer; *P* = .27).  No difference was found between the print and online flyers in terms of the memory measures  Recall: *P* = .86  Recognition: *P* = .16  Ad memory: *P* = .54 | None |

Abbreviations: N, number; NR, not reported; PC, personal computer; PDF, Portable Document Format; RQ, research question; SD, standard deviation; TV, television; URL, Uniform Resource Locator; US, United States.
